# Chitinase-like proteins promoting tumorigenesis through disruption of cell polarity *via* enlarged endosomal vesicles

**DOI:** 10.3389/fonc.2023.1170122

**Published:** 2023-04-28

**Authors:** Dilan Khalili, Martin Kunc, Sarah Herbrich, Anna M. Müller, Ulrich Theopold

**Affiliations:** Department of Molecular Biosciences, The Wenner-Gren Institute, Stockholm University, Stockholm, Sweden

**Keywords:** Drosophila, immunity, tumor, endosomal vesicles, salivary glands, chitinase, insect immunity

## Abstract

**Introduction:**

Chitinase-like proteins (CLPs) are associated with tissue-remodeling and inflammation but also with several disorders, including fibrosis, atherosclerosis, allergies, and cancer. However, CLP’s role in tumors is far from clear.

**Methods:**

Here, we utilize *Drosophila melanogaster* and molecular genetics to investigate the function of CLPs (imaginal disc growth factors; Idgf’s) in *Ras^V12^* dysplastic salivary glands.

**Results and discussion:**

We find one of the Idgf’s members, *Idgf3*, is transcriptionally induced in a JNK-dependent manner via a positive feedback loop mediated by reactive oxygen species (ROS). Moreover, *Idgf3* accumulates in enlarged endosomal vesicles (EnVs) that promote tumor progression by disrupting cytoskeletal organization. The process is mediated *via* the downstream component, aSpectrin, which localizes to the EnVs. Our data provide new insight into CLP function in tumors and identifies specific targets for tumor control.

## 1 Introduction

Chitinase-like protein (CLPs), including human YKL-39 and YKL-40 are synthesized and secreted under various conditions, including tissue injury, inflammatory and regenerative responses. Under pathological conditions they may contribute to asthma, sepsis, fibrosis and tumor progression (1, 2) including ductal tumors, such as the lung, breast, and pancreas (3, 4). CLPs are regulated by growth factors, cytokines, stress and the extracellular matrix (ECM). However, the causal connection between CLPs’ function and disease progression is only partially elucidated (5).

Animal models have been increasingly used in molecular oncology. This includes the fruitfly *Drosophila melanogaster*, where overexpression of dominant-active Ras (Ras^V12^) in proliferating tissue leads to benign tumors and simultaneous reduction of cell polarity genes to progression towards an invasive stage. (6–9). Central to this switch towards increasing malignancy is the C-Jun N-terminal kinase (JNK)-signaling pathway, which becomes activated *via* loss of cell polarity and promotes tumor growth (10). However, the outcome of activated JNK is mediated in a context-dependent manner due to downstream effects several of which are yet to be elucidated (11, 12). Among potential JNK regulators, spectrin family members belong to cytoskeletal proteins which form a spectrin-based membrane skeleton (SBMS) (13). Through the Rac family of small GTPases, cell polarity and SBMS organization are maintained (14, 15). Although the exact relationship between Spectrin and JNK in tumors remains to be established, Rac1 under physiological conditions cooperates with JNK in tissue growth (16–18).

To explore CLPs’ tissue autonomous function in a ductal tumor, we utilize the *Drosophila melanogaster* salivary glands (SGs). Generally, *Drosophila* CLPs are endogenously expressed in the larvae and include six members, termed Idgf 1-6 (Imaginal disc growth factors), that are involved in development, establishment of the cuticle, wound healing and restoration of cell organization (19–23). The SGs’ epithelial luminal organization and the conserved activation of the tumor-promoting signaling factors make them suitable for dissecting CLP function. Moreover, the lumen separating a single layer of cells can be disrupted by constitutive active *Drosophila Ras* (*Ras^V12^*) (24) leading to the loss of ECM integrity, the formation of fibrotic lesions and of the loss of secretory activity (25).

Here we investigated the role of *Drosophila* Idgf’s in *Ras^V12^*-expressing SGs. We show that one of the CLP’s members, *Idgf3*, is induced in tumor glands, leading to a partial loss of epithelial polarity and promoting a reduction of lumen size. The mechanism is driven through JNK signaling upstream of *Idgf3*. In line with previous work, ROS production *via* JNK mediates induction of *Idgf3*, creating a tumor-promoting signaling loop. Idgf3 further promotes the formation of enlarged endosomal vesicles (EnVs) *via* αSpectrin. Inhibiting EnVs formation by individually knocking-down *Idgf3* and *αSpectrin*, restores cell organization. Similar effects are observed upon expression of human CLP members in *Ras^V12^* SGs. Thus, our work identifies a phylogenetically conserved contribution of tumor-induced CLP’s towards the dysplasia of ductal organs and supports a role for spectrins as tumor modifiers.

## 2 Materials and methods

### 2.1 *Drosophila* maintenance and larvae staining

Stocks were reared on standard potato meal supplemented with propionic acid and nipagin in a 25°C room with a 12 h light/dark cycle. Female virgins were collected for five days and crossed to the respective males (see supplementary cross-list) after two days. Eggs were collected for six hours and further incubated for 18 h at 29°C. 24 h after egg deposition (AED), larvae were transferred to a vial containing 3 mL food supplemented with antibiotics (see **Supplementary Table S1**). 96 h and 120 h after egg deposition (AED), larvae were washed out with tap water before being dissected.

### 2.2 Sample preparation and immunohistochemistry

SGs were dissected in 1 x phosphate-buffered saline (PBS) and fixed in 4% paraformaldehyde (PFA) for 20 min. For extracellular protein staining, the samples were washed three times for 10 min in PBS and with PBST (1% TritonX-100) for intracellular proteins. Subsequently, samples stained for H2 were blocked with 0.1% bovine serum albumin (BSA) in PBS, and SG stained for pJNK, Idgf3, Spectrin, Dlg, p62 (ref(2)P), and GFP were blocked with 5% BSA for 20 min. After that, samples were incubated with the respective primary antibodies. Anti-pJNK (1:250), anti-Idgf3 (0.0134 µg/ml), anti-Spectrin (0.135 µg/ml) diluted in PBST were incubated overnight 4°C. anti-GFP (1 µg/ml) in PBST, H2 (1:5), and anti-SPARC (1:3000) in PBS were incubated for one hour at room temperature (RT). Samples were washed three times with PBS or PBST for 10 min and incubated with secondary antibody anti-mouse (4 µg/ml, Thermofisher #A11030) or anti-rabbit (4 µg/ml, Thermofisher #A21069) for one hour at RT. Subsequently, samples were washed three times in PBS or PBST for 10 min and mounted in FluoromountG.

### 2.3 Salivary gland size imaging and analysis

SG samples were imaged with Axioscope II (Objective 4x) (Zeiss, Germany) using AxioVision LE (Version 4.8.2.0). The images were exported as TIF and analyzed in FIJI (ImageJ: Version 1.53j). Representative confocal pictures were selected for figure panels and the complete set of replicate figures processed further for quantification (see below). Region of Interest (ROI) were drawn with the Polygon selection tool, and the scale was set to pixels (Px). The SG area was summarized as a boxplot with whisker length min to max. The bar represents the median. Statistical analysis was done with Prism software (GraphPad Software, 9.1.2, USA), the population was analyzed for normality with D’Agostino-Pearson and p-value quantified with Student’s t-test.

### 2.4 Nuclear volume imaging and quantification

Nuclei were stained with DAPI (1 µg/ml, Sigma-Aldrich D9542) in PBST for 1 h at RT. Mounted glands were imaged with Zeiss LSM780 (Zeiss, Germany) using a plan-apochromat 10x/0.45 objective with a pixel dwell 3.15 µs and 27 µm pinhole in z-stack and tile scan mode. Zeiss images were imported into ImageJ and viewed in Hyperstack. The selection threshold was set individually for each sample, and the analysis was performed with 3D objects counter. The nuclei volume was presented in boxplot, whisker length min to max and bar represent median. P-value quantified with Student’s t-test and the scale bar represent µm^3^.

### 2.5 Intensity and hemocyte quantification

The images for quantifying pJNK, TRE, Idgf3, and SPARC intensity and hemocyte recruitment were captured with AxioscopeII (Objective 4x) (Zeiss, Germany). The images were exported as TIF and analyzed in ImageJ. ROI was drawn with the Polygon selection tool, and subsequently, the total intensity was measured (pixel scale). The intensity was quantified according to the equation: Integrated Density – (SG area*Mean gray value). Hemocyte area was selected with Threshold Color and quantified by using the following equation: Ln (Hemocyte area + 1)/Ln (SG size + 1). Representative images were taken with Zeiss LSM780 (Zeiss, Germany). The images were then processed using Affinity Designer (Serif, United Kingdom). Graphs and statistical analysis were generated with Prism software (GraphPad Software, 9.1.2, USA). The population was analyzed for normality with D’Agostino-Pearson. Statistical significance was determined with Student’s t-test, One-way ANOVA with Tukey’s multiple comparison, and two-way ANOVA with Dunnett’s multiple comparison.

### 2.6 Enlarged endosomal vesicles penetrance quantification

The penetrance of the enlarged vesicles was subjectively quantified based on positive actin staining. Samples were analyzed in Axioscope II (Objective 20x) (Zeiss, Germany). At least 15 samples were analyzed with three independent replicates.

### 2.7 Humanized transgenic *Drosophila* lines

Plasmids were generated and transformed at VectorBuilder (https://en.vectorbuilder.com/). Human *CH3L1* and *CH3L2* genes were inserted into *Drosophila Gene Expression Vector pUASTattB* vector generating VB200527-1248haw and VB200518-1121xyy, respectively and transformed into *E. coli.* The bacteria were cultured in 3 ml LB supplemented with ampicillin (AMP: 100 ug/ml) for 15 h, at 37°C. The plasmid was extracted according to the GeneJetTm Plasmid Miniprep Kit #K0503 standard procedure. Plasmids were validated through sequencing at Eurofins (https://www.eurofins.se/: For primer details see **Supplementary Table S1**). *Drosophila* transgenic lines were generated at thebestgene (https://www.thebestgene.com/). Plasmids were extracted with QIAGEN Plasmid Maxi Kit according to the standard procedure and injected into *w^1118^* strains. Expression of the human CLPs was validated with qPCR.

### 2.8 *In situ* hybridization

The Idgf3 (GH07453: DGRC) probe was generated according to (26). The staining procedure is described elsewhere with the following changes (26). The procedure was conducted in 200 µl transwells containing four salivary glands. The procedure included three technical replicates per genotype. Images were aqured with Leica MZ16 (Leica, Germany) microscope and Leica DFC300x FX digital color camera (Leica, Germany). Representative images were taken, and figures were generated in Affinity Designer (Serif, United Kingdom).

### 2.9 qPCR

mRNA isolation and cDNA synthesis were performed according to manufacture instructions (AM1931). qPCR procedures were performed as described earlier (24) with an adjusted Kappa concentration to 0.5x. At least three replicates and two technical replicates were performed for each qPCR. See **Supplementary Table S1** for primer list.

## 3 Results

### 3.1 Idgf3 promotes a dysplastic phenotype

**Figure 1:**
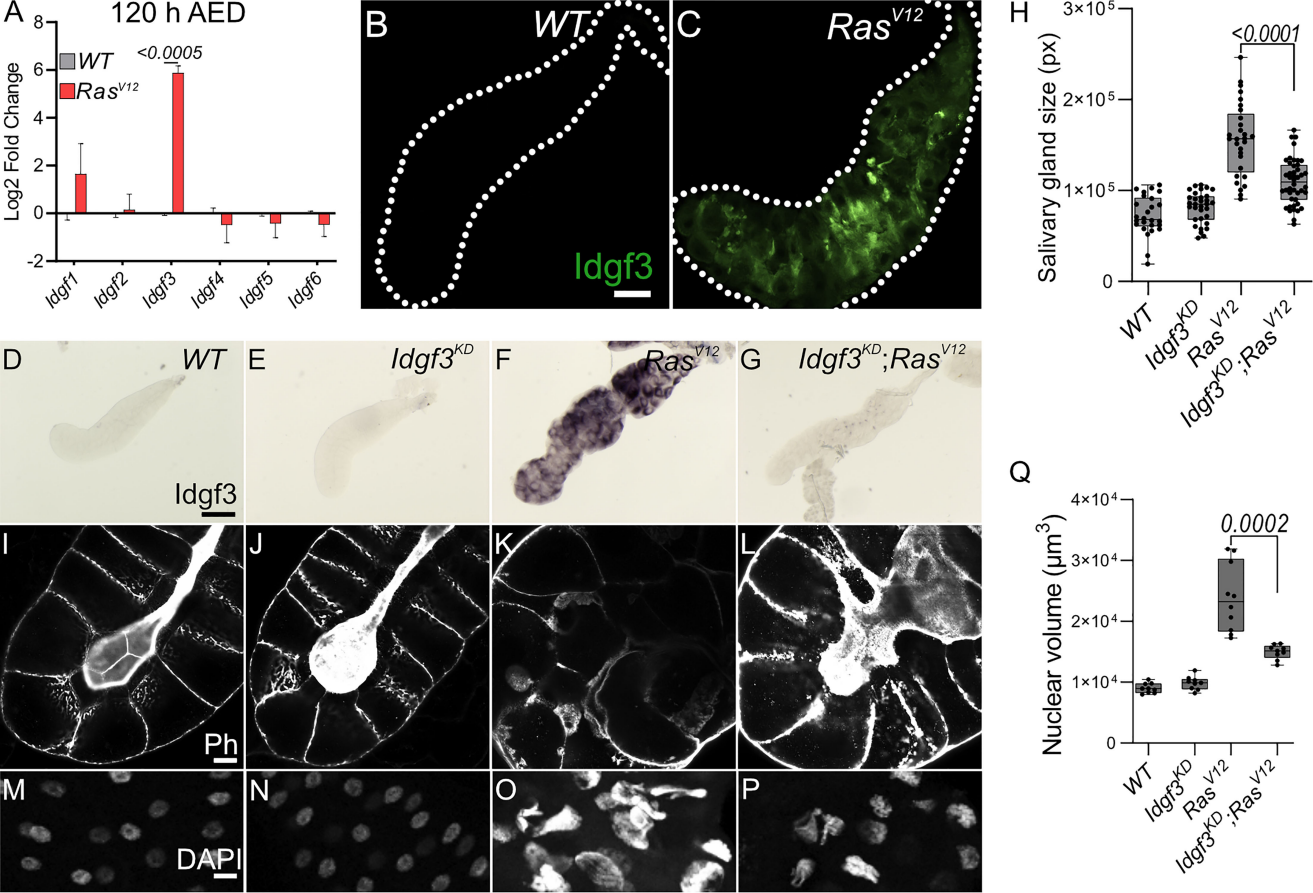
Idgf3 promotes growth and disrupts tissue architecture **(A)** qPCR data showing induction of *Idgf3* in 120 h AED *Ras^V12^* glands. **(B, C)** Idgf3 tagged with GFP was localized in the dysplastic glands. **(D-G)** Knock-down of *Idgf3* in *Ras^V12^* glands confirmed reduced mRNA levels as shown by *in situ* hybridization. **(H)** SG size quantification showing a reduction in tissue size in *Idgf3^KD^;Ras^V12^* SG compared to *Ras^V12^* alone. **(I-L)** F-actin (Phalloidin) staining revealed partial restoration of the lumen in *Idgf3^KD^;Ras^V12^* glands, in comparison to *Ras^V12^* alone. **(M-P)** Nuclei in DAPI stained SG displayed a reduced size in *Idgf3^KD^;Ras^V12^;* quantified in **(Q)**. Scale bars in **(B, C)** represent 100 µm, **(D-G)** represent 0.3 mm and **(I-L)** represent 20 µm **(M-P)** represents 10 µm. Data in **(A)** represent 3 independent replicates summarized as mean ± SD. Boxplot in **(H, Q)** represent at least 20 SG pairs. Whisker length min to max, bar represent median. P-value quantified with Student’s t-test.

Obstruction of SG lumen by the constitutive-active oncogene, *Ras^V12^*, under *Beadex-Gal4* driver (*Ras^V12^*) disrupts organ function between 96 h and 120 h after egg deposition (AED) (25). Being that CLPs have been implicated in the loss of cell polarity (27), we investigated whether *Drosophila* CLPs contribute to the observed phenotype. First, to find out whether CLPs were induced in the *Ras^V12^* glands, we assessed relative mRNA levels at two different time points, 96 h and 120 h AED. Only one of the *CLP* members, namely *Idgf3*, was significantly upregulated at both time points (**Figures 1A**, **S1A**). Therefore, we decided to focus on *Idgf3’s* effects on dysplastic glands.

Idgf3 contains an N-terminal signal peptide and has been detected in hemolymph (28). To analyze its subcellular tissue distribution in SGs, we used a C-terminally GFP tagged version of Idgf3 (21). At first we used *in situ* hybridization to show distribution of Idgf3 in salivary glands (**Figures S1B-E**). At 96 h we could not detect Idgf3 in the whole *WT* or *Ras^V12^* animals (**Figures S1F-G’**), possibly due to limited sensitivity. Likewise, 120 h old *WT* larvae did not show any detectable signal (**Figure S1H-H’**) while a strong Idgf3 signal was detected in *Ras^V12^* SGs (**Figure S1I-I’**). To better understand Idgf3 distribution at a higher resolution, we dissected 120 h AED glands. *WT* glands had a weaker Idgf3::GFP signal in comparison to the *Ras^V12^* (**Figures 1B, C**). Moreover, Idgf3 was unevenly distributed throughout *Ras^V12^* SGs (**Figure 1C**).

The increased level of *Idgf3* between 96 h and 120 h strongly correlated with loss of tissue- and cell-organization and an increased nuclear volume (24). In order to characterize the role of Idgf3 in *Ras^V12^* glands, we used a specific *Idgf3 RNA-interference* line (*Idgf^KD^*). Moreover, we focused on 120 h larvae, unless otherwise stated, since they showed the most robust and developed dysplastic phenotype. Efficient knockdown of *Idgf3* was confirmed using *in situ* hybridization and at the protein level (**Figures 1D-G**, **S1J-M**; quantified in N, (21)). Macroscopic inspection showed that *Idgf^KD^;Ras^V12^* SGs were smaller than *Ras^V12^* SGs (**Figure 1H**), resembling WT controls. To gain insight into the cellular organization, we stained the glands for F-actin (Phalloidin: Ph) and DNA using DAPI. In *Idgf^KD^* the cells retained their cuboidal structure, and the lumen was visible as in *WT*, indicating that Idgf3 on its own does not affect apicobasal polarity (**Figures 1I, J**). In contrast, in *Ras^V12^* glands apicobasal polarity was lost, and the lumen was absent (**Figures 1K**, (25)). In *Idgf^KD^;Ras^V12^* SGs a reversal to the normal distribution of F-actin and partial restoration of the lumen was observed (**Figure 1L**). Similarly, the nuclear volume, which increased in *Ras^V12^* SGs returned to near wild type levels upon *Idgf^KD^* (**Figure 1M-P**, quantified in **Figure 1Q**). This indicates that *Idgf^KD^* can rescue Ras^V12^-induced dysplasia.

In order to unravel the specific effects mediated by Idgf3 we further investigated *Ras^V12^* associated phenotypes, including fibrosis and the cellular immune response. As recently reported, *Ras^V12^* SGs displayed increased levels of the extracellular matrix components (ECM), including collagen IV and SPARC (BM40, (25)). *Idgf^KD^* did not affect SPARC levels in comparison to the *WT* (**Figures S1O-P**) but *Idgf-KD;Ras^V12^* SGs displayed significantly reduced SPARC levels in comparison to *Ras^V12^* (**Figures S1Q-R**, quantified in S). To assess whether this led to a reduced inflammatory response, we investigated the recruitment of plasmatocytes, macrophage-like cells previously reported to be recruited towards tumors (9). We found that both control and *Idgf^KD^* glands did not show recruitment of hemocytes (**Figures S1T-U**). In contrast to the effects on ECM components, *Idgf^KD^ in Ras^V12^* glands did not lead to any changes in hemocyte attachment (**Figures S1V-W**, quantified in X). Taken together, upon *Ras^V12^* overexpression, Idgf3 promotes SG overgrowth, loss of cell organization, and fibrotic-like accumulation of the ECM, but not immune cell recruitment.

### 3.2 Idgf3 induces dysplasia *via* JNK-signaling

Dysplasia is driven by internal and external factors that either work in concert or independently. Similar to what we observed in *Idgf^KD^;Ras^V12^* glands blocking the sole *Drosophila* JNK member *basket* reverts many tumor phenotypes (24). Moreover, the dysplastic loss of apical and basolateral polarity between 96 h and 120 h is driven by the JNK-pathway (24). The time frame when we observed upregulation of *Idgf3* (**Figures 1A**, **S1A**) coincides with the period during which blocking JNK restores tissue organization and homeostasis, similar to what occurs in *Idgf^KD^;Ras^V12^* tissues (**Figures 1L**, **S1R**). Therefore, we decided to test a possible involvement of JNK-signaling in the regulation of Idgf3.

**Figure 2:**
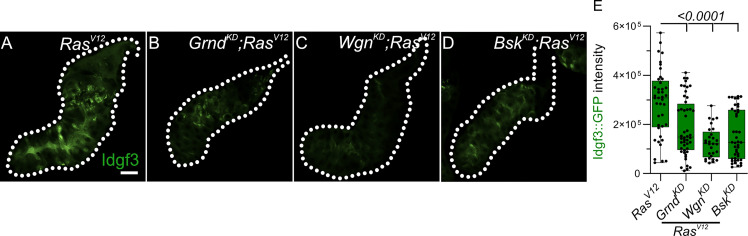
Idgf3 dysplasia is mediated through JNK activity **(A-D)** Representative images of Idgf3::GFP in a JNK targeted screen. **(E)** Quantification showing Idgf3::GFP intensity was reduced by *Grnd^KD^*, *Wgn^KD^* and *Bsk^KD^* in *Ras^V12^* SG. Scale bars in **(A-D)** represent 100 µm. Boxplot in **(E)** represents at least 20 SG pairs. Whisker length min to max, bar represent median. P-value quantified with Student’s t-test.

First, we performed a targeted JNK RNAi-screen using Idgf3::GFP intensity in the glands as readout upon KD of JNK signaling components. We first confirmed the sensitivity of the Idgf3::GFP construct by *Idgf3-KD* in *Ras^V12^* SGs compared to *Ras^V12^* glands (**Figures S2A, B**, quantified in **Figure S2C**). Knockdown of the two classical TNF receptors upstream of JNK, *Grnd* (*Grindelwald*) and *Wgn* (*Wengen*) (**Figure S2D**) similarly reduced Idgf3::GFP intensity (**Figures 2A–C**, quantified in **Figure 2E** (29). Similar effects were observed with *Bsk^KD^* (**Figure 2D**, quantified in E). Altogether this suggests that Idgf3 protein levels are regulated downstream of JNK and the TNF members *Grnd* and *Wgn*.

### 3.3 ROS promotes Idgf3 induction *via* JNK

**Figure 3:**
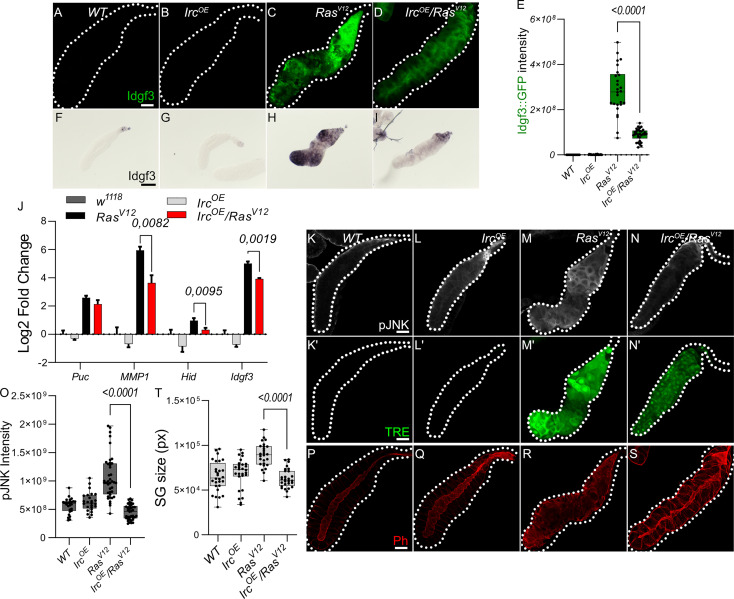
Antioxidants overexpression reduce Idgf3 and JNK signaling activity **(A-D)** Reduction of H_2_O_2_ by overexpression of secreted catalase (immune regulated catalase; IRC) lowered Idgf3::GFP levels, quantified in **(E)**. **(F-I)** ISH showing reduced expression of *Idgf3* in *IRC-OE;Ras^V12^* glands. **(J)** qPCR data showing reduction of *Idgf3, puc*, *MMP1* and *Hid* in *IRC^OE^;Ras^V12^* glands. Selection of JNK targets with the most significant induction was partially based on our previous experiments ((24), **Figure, S3A**). **(K-N’)** pJNK staining and TRE reporter constructs showing reduced intensity in *IRC^OE^;Ras^V12^* in comparison to *Ras^V12^* glands, quantified in **(O)**. **(P-S)** Phalloidin staining showing partially restored lumen in *IRC^OE^;Ras^V12^* glands, quantified in **(T)**. Scale bars in **(A-D, K-S)** represent 100 µm and **(F-I)** represent 0.3 mm. Data in **(J)** represent 3 independent replicates summarized as mean ± SD. Boxplot in **(E, O, T)** represent at least 20 SG pairs. Whisker length min to max, bar represent median. P-value quantified with Student’s t-test.

To further dissect Idgf3 regulation, we focused on the positive JNK regulators, reactive oxygen species (ROS) both intra- and extracellularly (9, 30). We previously reported that ROS production in *Ras^V12^* SGs increases *via* JNK (24). To inhibit ROS intra- and extracellularly, we separately overexpressed the H_2_O_2_ scavengers Catalase (Cat) and a secreted form of Catalase, IRC (immune-regulated Catalase), and ${\text{O}}_{2}^{-}$ scavenger SOD (Superoxide dismutase A), in the *Ras^V12^* background and quantified Idgf3::GFP intensity. Reducing levels of intracellular H_2_O_2_ (*Cat^OE^*), but not ${\text{O}}_{2}^{-}$ (*SOD^OE^*) lowered Idgf3::GFP intensity (**Figures S3A-D** quantified in E). Similarly, reduction of extracellular H_2_O_2_ by the secreted version of Catalase (Irc^OE^) lowered Idgf3::GFP levels (**Figures 3A-D**, quantified in **Figure 3E**) as well as JNK signaling (**Figures 3J, K-N’**, quantified in **Figure 3O**). We used detection of pJNK and TRE-GFP1b reporter construct, which recapitulates JNK-activation by expressing GFP under control of biding sites for JNK-specific AP-1 transcription factors (31). Confirming JNK-activation, three known JNK targets (puckered, *puc*, a negative feedback regulator of JNK; metalloproteinase 1, *MMP1* and head involution defective, hid (32–34)) as well as Idgf3 itself showed the same dependence on Irc^OE^. In line with the reduced tissue size and improved tissue integrity in *Idgf3^KD^;Ras^V12^*, overexpression of *IRC* in *Ras^V12^* SGs also reduced SG size (**Figure 3T**), improved tissue integrity and restored the SG lumen (**Figure 3F-I, P-S**).

In summary, ROSs contribute to pJNK signaling. In addition, overexpression of extracellular and intracellular Catalase but not SOD reduces Idgf3 induction *via* JNK, similar to the feedback loop that has been identified in other tumor models (9).

### 3.4 Idgf3 accumulates in large vesicles, which display markers for endocytosis and macropinocytosis

**Figure 4:**
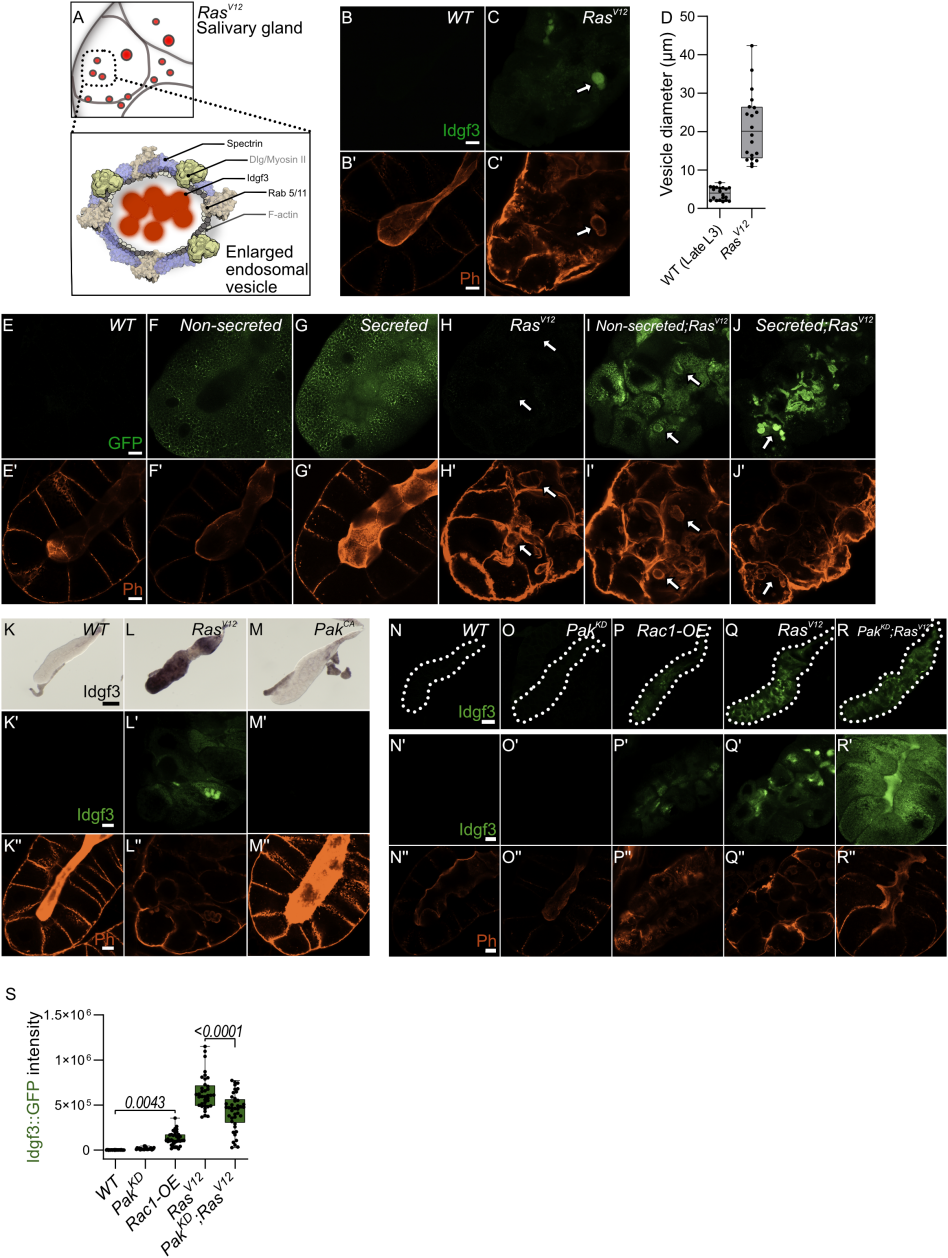
*Idgf3* promotes formation of enlarged endosomes in the Ras^V12^ background **(A)** The overall figure shows Ras^V12^ salivary gland with EnVs marked by red color. The inlet depict Idgf3 enclosed by enlarged vesicles (EnVs) coated by cytoskeletal and cell polarity proteins. **(B-C’)** Idgf3::GFP clusters coated with Phalloidin. **(D)** Vesicle size quantification showing *Ras^V12^* enlarged vesicles in comparison to prepupae SG vesicles. **(E-J’)** Non secreted MFGE8 localizes to the surface of EnVs, co-stained with phalloidin. The secreted MFGE8 is packaged into EnVs in *Ras^V12^* glands. **(K-M)** ISH showing no induction of *Idgf3* by constitutively active Pak. **(K’-M’’)** Idgf3::GFP is not detectable in the *Pak^CA^* glands and the lumen is detectable (Phalloidin). **(N-R’’)** Restoration of the lumen packed with Idgf3::GFP, no formation of EnVs and Idgf3 in *Pak^KD^;Ras^V12^* glands. Scale bars in **(B-C’, E-J’, K’-M’’, N’-R’’)** represent 20 µm, **(K-M)** represents 0.3 mm and **(N-R)** represents 100 µm. Boxplot in **(D)** represents 20 EnVs and **(S)** represents at least 20 SG pairs. Whisker length min to max, bar represent median. P-value quantified with Student’s t-test.

We previously noted the uneven distribution of Idgf3 in *Ras^V12^* SGs (**Figure 1C**). To further understand how Idgf3 promotes dysplasia, we dissected its subcellular localization (**Figure 4A**). We stained the glands for F-actin (Phalloidin) and addressed Idgf3::GFP localization at high resolution (**Figures 4B, C’**). Interestingly, we observed Idgf3::GFP clusters surrounded by F-actin (**Figures 4C-C’**: arrow). Using a different salivary gland driver (*AB-Gal4*) to drive expression of *Ras^V12^*, we also observed increased expression of Idgf3::GFP and its localization within vesicular structures (**Figure S4A-B’**: arrow). The size of the vesicle-like structures was between 10-43 µm in comparison to secretory *Drosophila* vesicles (3-8µm, **Figure 4D**) (35). We refer to these as enlarged vesicles (EnVs). Based on the increased Idgf3 levels, we wondered whether the protein was aggregating in EnVs. The aggregation marker p62, which is autophagic adaptor marking cytoplasmic protein aggregates prepared for clearance (36) was strongly bound to the cytoplasm of *Ras^V12^ SGs* unlike from *WT* glands. However, the EnVs did not contain any aggregated proteins (**Figure S4C-D’**). This may imply that Idgf3 is even taken up from the SG lumen in a soluble state.

Since we had observed a loss of secretion in *RasV12* SGs we next addressed the presence of EnVs within the secretory pathway. We overexpressed two versions of human phosphatidylserine binding protein, MFG-E8 (Milk fat globule-EGF factor), without (referred as non-secreted: **Figures 4F, F’, I, I’**) and with a signal peptide (referred as secreted: **Figures 4G, G’,J, J’** 37). In controls, the non-secreted form was found in the cytoplasm, whereas the secreted version was detected in the cytoplasm and in the lumen (**Figures 4E–E’, F–G’**). In *Ras^V12^* SGs, the non-secreted form was surrounding the EnVs (arrow), indicating the presence of phosphatidylserine on their membrane (**Figures 4H–H’, I, I’**). In contrast, the secreted form localized to the inside of the EnVs (**Figures 4J, J’**: arrow). These data suggest that EnVs are surrounded by a lipid membrane and probably derive from the secretory pathway.

In order to further characterize Idgf3-containing EnVs we co-expressed vesicle-specific Rab’s coupled with a GFP fluorophore, a lysosomal marker (Atg8), an autophagy marker (Vps35), and a marker for phosphatidylinositol-3-phosphate-(PtdIns3P: *FYVE*)-positive endosomes in *Ras^V12^* glands (For a complete set, see **Figure S4E-M’’**) which marks macroautophagy vesicles. To increase sensitivity and to identify EnVs, we stained with anti-GFP and co-stained with Phalloidin. Localization of Rabs and phalloidin to the same vesicles was observed with endosomal marker (Rab5) and recycling endosomal marker (Rab11) but not endosomal marker (Rab7) (**Figure S4E-H’’, S4M-M’’**). Moreover, EnVs were also positive for PtdIns3 (**Figure S4L-L’’**). In line with their dependence on secretion, this potentially identifies EnVs as enlarged recycling endosomes. EnV accumulation in *Ras^V12^* glands between 96 h and 120 h implies that (i) endosome formation is either increased compared to *WT* or (ii) that endosomes are not normally recycled leading to their accumulation. The latter hypothesis correlates with the loss of apico-basolateral polarity and the disruption of secretion due to a lack of a luminal structure in *Ras^V12^* glands (25). To test the first hypothesis, we blocked the formation of early endosomes with *Rab5^DN^*. Apico-basolateral polarity, detected by a visible lumen, was not affected by *Rab5^DN^*. Moreover, *Rab5^DN^;Ras^V12^* did not block EnV formation and restoration of apicobasal polarity (**Figure S4N-Q**). Halting the recycling endosome pathway *via Rab11^DN^* increases the endosomes’ accumulation without affecting cell polarity (**Figure S4R**). In contrast, in *Rab11^DN^;Ras^V12^* SGs, endosomes were not accumulating, and EnVs were still detected (**Figure S4S**). Taken together, EnV formation is independent of the classical recycling pathway, suggesting other candidates are involved in their generation.

In SGs, overexpression of Rac generates enlarged vesicles with similarity to the EnVs described here (14). Supporting a role in dysplasia in our system, *Ras^V12^* SGs showed stronger Rac1 expression in comparison to the control. Due to the pleiotropic effects of the *Rac1^DN^* construct, we addressed Rac1 function by modulating the expression of the Rac1 effector molecule, *Pak* (14). Overexpression of *Pak^CA^* did not increase *Idgf3* levels and had no detectable effect on F-actin distribution (**Figures 4K-M’’**). In contrast, Rac1 activity *via* Pak does affect *Ras^V12^* SG integrity: Idgf3::GFP levels were increased in *Rac1-OE* SGs and F-actin was disorganized (**Figures 4N-P’’** quantified in **S**). However, the *Rac1-OE* glands did not grow larger compared to *Ras^V12^*,indicating additional signals are necessary for gland overgrowth. Also, *Pak^KD^;Ras^V12^* SGs displayed a more regular F-actin distribution leading to restoration of the lumen and proper secretion of Idgf3 (**Figures 4Q-R’’** quantified in S). Moreover, we observed Rac1 also localized to EnVs (**Figure S4T-U’**). Decoration with Rac1 and actin as well as their dependence on Ras activation potentially identifies EnVs as macropinocytotic vesicles ((38), see also discussion). The enlarged vesicles that form upon Rac overexpression in SGs (14) also stain positive for Spectrins identifying them as additional candidates for EnVs formation. Of note, Spectrins under physiological settings are involved in the maintenance of cellular integrity including epithelial organization, which is lost in *Ras^V12^* SGs.

### 3.5 JNK promotes EnVs formation *via* Idgf3 upstream of αSpectrin

**Figure 5:**
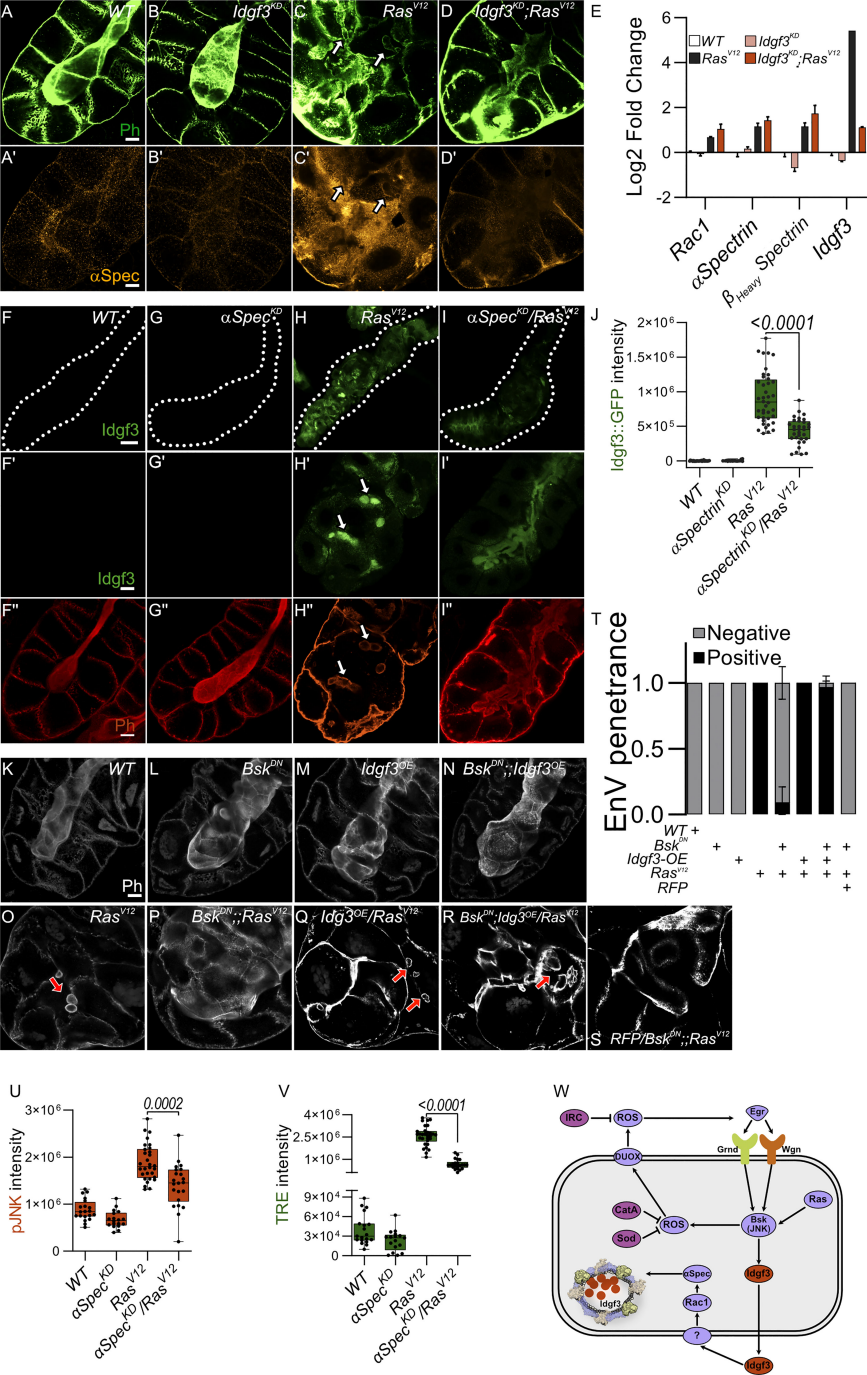
JNK promotes EnVs formation *via* Idgf3 upstream of αSpectrin **(A-D’)** αSpectrin staining showing restoration of normal distribution in *Idgf3^KD^;Ras^V12^* glands. **(E)** qPCR data showing no reduction of *αSpectrin* and *β_Heavy_Spectrin* in *Idgf3^KD^;Ras^V12^.* **(F-I’’)** Reduced levels of αSpectrin (αSpectrin*^KD^* ^/^ *Ras^V12^*) reduces Idgf3::GFP levels quantified in **(J)**, prevents formation of EnVs and largely restores the SG lumen (arrows indicate EnVs). **(K-S)** Phalloidin staining showing epistasis of EnVs formation in which Idgf3 acts downstream of JNK. **(T)** EnVs penetrance quantification showing a strong induction of EnVs in *JNK^DN^;Idgf3^OE^/Ras^V12^* glands. **(U)** pJNK intensity quantification showing reduced levels in *aSpectrin^KD^/Ras^V12^.* **(V)** TRE intensity quantification showing reduced levels in *aSpectrin^KD^/Ras^V12^.* **(W)** Idgf3 promotes formation of EnVs, upstream of Rac1. Scale bars in **(A-D’, F’-I’’, K-S’)** represent 20 µm, **(F-I)** represents 100 µm. Data in **(E)** represent 3 independent replicates summarized as mean ± SD. Barplot in **(T)** represent 3 independent replicates with at least 10 SG pairs, summarized as mean ± SD. Boxplot in **(J, U, V)** represent at least 20 SG pairs. Whisker length min to max, bar represent median. P-value quantified with Student’s t-test.

To analyze Spectrin contribution to EnVs formation, we stained for αSpectrin, one of the three members in flies (39) and found it to be induced in *Ras^V12^* SGs and to localize to the EnVs (**Figure S5A-B’’**). Knockdown of *Idfg3* in *Ras^V12^* SGs reduced both αSpectrin levels and EnVs formation (**Figures 5A-D’**). Despite efficient *Idfg3^KD^*, transcript levels for both α- and β_Heavy_Spectrin as well as for Rac1 were not affected indicating regulation at the posttranscriptional level (**Figure 5E**). Moreover, we found markers for cell polarity including Dlg, and Myosin II also decorate the EnVs (**Figure S4V-Y’**: arrow). In contrast, αSpectrin^KD^ (**Figure S5C-F** quantified G) reduced Idgf3 levels (**Figures 5F-I’** quantified **Figure 5J**) as well as JNK signaling upstream of Idgf3 (**Figures 5U, V**). Further supporting a role for Spectrins in SG dysplasia, knockdown of αSpectrin in *Ras^V12^* glands abolished EnVs formation and partially restored the SG lumen (**Figure 5I’’**).

Taken together this suggests that Idgf3 promotes EnVs formation (**Figures 5C, C’**) most likely post-transcriptionally (**Figure 5E**). In line, overexpression of Idgf3 throughout the whole gland, at 96 h, as shown by ISH (**Figure S5I-L**), led to an increase in the number of glands with endosomes (**Figure S5M-P’’’**, quantified in Q). To address epistasis between Idgf3 and JNK we calculated the penetrance of EnVs formation. In *Ras^V12^* SGs we observed EnVs in 100% of the glands, an effect that was strongly blocked in *Bsk^DN^;Ras^V12^* (**Figures 5O, S**, quantified in **Figure 5T**). Blocking JNK and overexpressing *Idgf3* in *Ras^V12^* strongly reverted the *Bsk^DN^;Ras^V12^* phenotype, a lumen could not be detected, and around 98% of the glands contained enlarged endosomes (**Figures 5O–S** quantified in **Figure 5T**) while control SGs using RFP-overexpression retained the *Bsk^DN^;Ras^V12^* phenotype. Overexpression of Idgf3 alone did not result in EnVs formation (**Figures 5K–N**). In conclusion, the data suggest that Idgf3 acts downstream of JNK and - through formation of EnV’s - disrupts luminal integrity. The proposed activity of Idgf3 in EnVs formation is summarized in **Figure 5W**.

### 3.6 Human CLP members enhance dysplasia in *Drosophila* SGs

**Figure 6:**
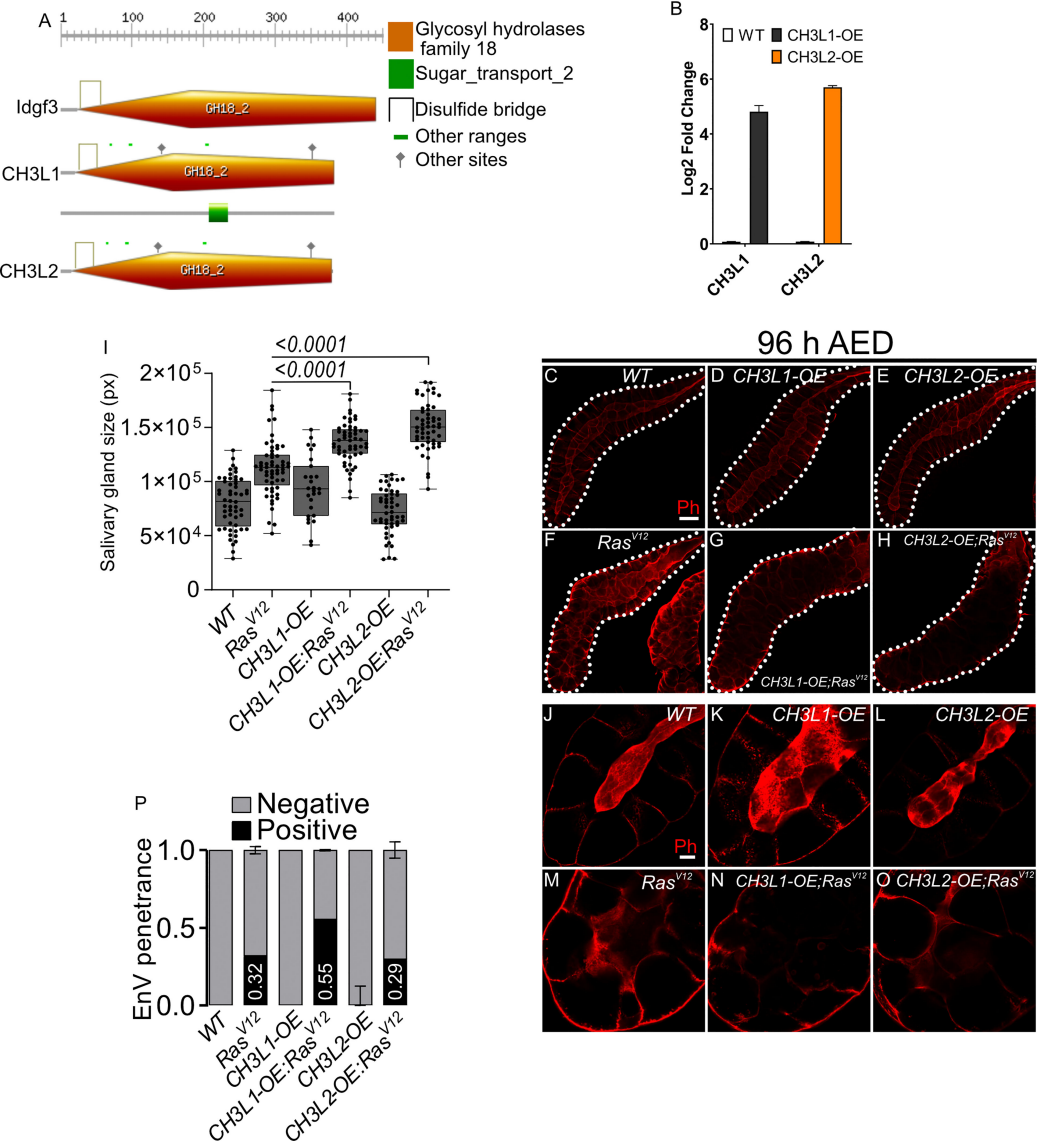
Human Chitinase-like proteins similarly to Idgf3 promotes EnVs formation **(A)** Comparison of Idgf3, CH3L1 and CH3L2 protein motifs (https://prosite.expasy.org). **(B)** qPCR confirmation of CH3L1 and CH3L2 expression in SG. **(C-H)** Representative images of phalloidin staining used for size quantification. **(I)** SG size quantification showing an increase in tissue size in *CH3L1^OE^;Ras^V12^* and *CH3L2^OE^;Ras^V12^* SG compared to *Ras^V12^* alone. **(J-O)** Phalloidin staining depicting disrupted lumen integrity in *Ras^V12^* glands. **(P)** EnVs penetrance quantification showing an induction of EnVs in *CH3L1^OE^;Ras^V12^* glands. Barplot in **(B)** represent 4 independent replicates with at least 10 SG pairs, summarized as mean ± SD. Scale bars in **(C-H)** represent 100 µm and **(J-O)** 20 µm. Boxplot in **(I)** represent at least 20 SG pairs. Whisker length min to max, bar represent median. P-value quantified with Student’s t-test. Barplot in **(P)** represent 3 independent replicates with at least 10 SG pairs, summarized as mean ± SD.

Finally, we wished to determine whether the tumor-modulating effects we had observed for *Drosophila* Idgf3 also applies to human CLP members. For this we expressed two human *CLPs* (*Ch3L1* or *Ykl-40*; 29% amino acid identity to Idgf3 and *Ch3L2* or *Ykl-39*; 26% amino acid identity, **Figure 6A**) in SGs, both on their own and in combination with *Ras^V12^*. Overexpression of CLPs in salivary glands was confirmed by qPCR (**Figure 6B**). Similar to Idgf3, both CLPs enhanced the hypertrophy observed in *Ras ^V12^* SGs (**Figures 6C-H** quantified in **Figure 6I**). The lumen integrity stayed highly disturbed when CLPs were overexpressed in Ras^V12^ background (**Figures 6J–O**). Additionally, *Ch3L1* enhanced the prevalence of EnVs in the Ras mutant background (**Figure 6P**). Taken together this means that the tumor-promoting effect of CLPs is conserved between *Drosophila* and humans and may affect different phenotypes of dysplasia depending on the CLP under study.

## 4 Discussion

The levels of Chitinase-like proteins (CLPs) are elevated during a wide range of inflammatory processes as well as neoplastic disorders. Their physiological function has been more elusive but includes the formation of extracellular assemblages (40) including the insect cuticle (22), wound healing and in both mammals (40) and insects (41) and the restoration of cell integrity after oxidative damage (42). Conversely, induction of CLPs has been associated with the development of fibrotic lesions and cancer development with poor prognosis (reviewed in (40)). We used *Drosophila* as a tumor model to dissect CLP (*Idgf3*) function genetically in a secretory ductal organ, the salivary glands. We show that Idgf3 promotes tumor overgrowth through the disruption of cell polarity. The induction of *Idgf3* disrupts cell organization and leads to the formation of enlarged endosome vesicles (EnVs) which accumulate in the cytoplasm. Genetically, *Idgf3* is induced *via* a pro-tumorigenic JNK and ROS signaling feedback loop. Consequently, Idgf3 recruits the spectrin-based membrane skeleton (SBMS) for the formation of EnVs. Significantly, KD of *Idgf3* inhibits overgrowth, restores cell polarity, reduces ECM size and blocks EnV formation.

Our identification of a contribution of JNK signaling and both extra- and intracellular ROS to dysplasia is in line with previous findings from other *Drosophila* tumor models (43). Similarly, like others (43) we observe an amplification loop between ROS and JNK signaling, which augments the dysplastic phenotype (24). Several studies have demonstrated that activation of JNK signaling in mammals promotes the progression of ductal tumors (44–46). Here we identify Idgf3 as an additional component that feeds into JNK signaling. Ultimately in Ras^V12^-expressing SGs this leads to the formation of EnVs involving Spectrins. Under physiological conditions, members of the Spectrin family have a supporting role in maintaining cellular architecture through interaction with phospholipids and actively promoting polymerization of F-actin (47–49). Moreover, the secretory activity of ductal organs has been shown to be facilitated by Spectrins (50).

During *Drosophila* development and under physiological conditions, the pathway that involves Spectrins, Rac1 and Pak1 has been shown to be required for the maintenance of cell polarity while when deregulated it leads to the formation of enlarged vesicles similar to the EnVs (14). Thus, our results provide a possible link between the observed induction of CLPs in a range of tumors and the effects of Spectrins and their deregulation in tumors (51, 52). In addition to the genetic interaction we find, previous work suggests an additional mechanical link *via* a Spectrin binding protein (Human spectrin Src homology domain binding protein1; Hssh3bp1, (53)) the loss of which has been associated with prostatic tumors (54). Hhh3bp1 may influence tumor progression possibly through interaction with tyrosine kinases such as Abelson kinase (54). Interestingly Hhh3bp1 is a marker and possible regulator of macropinocytosis (55), a recycling pathway that is known to be hijacked by Ras-transformed tumor cells to acquire nutrients (38) and also leads to the formation of large intracellular vesicles (56). In favor of this hypothesis macropinocytosis is known to depend on Rac1/Pak1 signaling although the resulting vesicles are usually smaller (0.2-5 micrometers) than EnVs (57). We find that - like macropinocytosis - EnV-formation depends on the activity of growth factors (38), in this case Idgf3, much in line with its original description as an *in vitro* mediator of insulin signaling (20). *In vivo*, under normal conditions Idgf3 is required for proper formation of chitin-containing structures, wound healing and cellular integrity (22). Thus, under these circumstances Idgf3 acts to preserve cellular integrity including the epithelial character of SG cells upstream of spectrins. We propose that in a non-physiological setting such as upon overexpression of *Ras^V12^*, this mechanism is overwhelmed leading to the breakdown of homeostasis, loss of cell polarity and the gland lumen, loss of secretory activity and the formation of EnVs larger than macropinocytotic vesicles. Large vesicles accompany several scenarios of non-apoptotic programed cell death, which occurs a.o. in apoptosis-resistant tumors (58, 59). Such modes of cell death include methuosis, a deregulated form of macropinocytosis (56, 58). Of note, apoptotic cell death is inhibited in *Drosophila* polytenic SGs to account for the increased number of DNA breaks that occur during endoreplication, which in mitotic cells induce apoptosis in both a p53-dependent and independent manner (60, 61). In line, despite the activation of caspase activity and nuclear fragmentation, which are considered hallmarks of apoptosis, *Ras^V12^* SG cells don’t disintegrate to produce apoptotic bodies (24). This may also explain the difference to mitotically cycling tumor models, which also activate JNK – yet with apoptosis as an outcome (32, 62, 63). Thus, SGs provide a suitable model for apoptosis-resistant tumors. In a mammalian setting, the phenotypes that are associated with non-apoptotic cell death such as disruption of cellular polarity and reorganization of the ECM provide potential targets for therapeutic treatments (46). Our work adds CLPs and spectrins to this list. Depending on the tissue environment and similar to JNK signaling, CLP’s may have varying roles in a context-dependent manner. Overexpression of *Idgf3* alone is not sufficient for the loss of cell polarity, overgrowth, and fibrosis. Collectively, this suggests a tumor-specific phenotype for Idgf3 (**Figures 6C-H**), in line with mammalian CLPs (reviewed in (40)). Due to their pleiotropic effects, further investigation of CLPs role will be required to dissect their molecular function in a given tissue and to ultimately design tumor-specific treatments (64).

Taken together our findings provide new insight into the loss of tissue integrity in a neoplastic tumor model including the contribution of CLPs, Spectrins and alternative forms of cell death. This may provide further ways to test how developmentally and physiologically important conserved mechanisms that maintain cellular hemostasis - when deregulated - contribute to tumor progression.

## Data availability statement

The original contributions presented in the study are included in the article/**Supplementary Material**. Further inquiries can be directed to the corresponding author.

## Author contributions

DK, UT and MK conceived the research and designed the experiments. DK, MK, SH and AM performed experiments and data analysation. DK, UT and MK wrote the paper and participated in the revisions. All authors contributed to the article and approved the submitted version.

## References

[1] Roslind A Johansen JS. YKL-40: a novel marker shared by chronic inflammation and oncogenic transformation. Methods Mol Biol (2009) 511:159–84. doi: 10.1007/978-1-59745-447-6_7 19347297

[2] Shao R Hamel K Petersen L Cao QJ Arenas RB Bigelow C. YKL-40, a secreted glycoprotein, promotes tumor angiogenesis. Oncogene (2009) 28:4456–68. doi: 10.1038/onc.2009.292 PMC2795793 19767768

[3] Johansen JS Jensen BV Roslind A Nielsen D Price PA. Serum YKL-40, a new prognostic biomarker in cancer patients? Cancer Epidemiol Biomarkers Prev (2006) 15:194–202. doi: 10.1158/1055-9965.EPI-05-0011 16492905

[4] Uhlen M Zhang C Lee S Sjostedt E Fagerberg L Bidkhori G. A pathology atlas of the human cancer transcriptome. Science (2017) 357(6352):357. doi: 10.1126/science.aan2507 28818916

[5] Park KR Yun HM Yoo K Ham YW Han SB Hong JT. Chitinase 3 like 1 suppresses the stability and activity of p53 to promote lung tumorigenesis. Cell Commun Signal (2020) 18:5. doi: 10.1186/s12964-019-0503-7 32127023 PMC7055043

[6] Brumby AM Richardson HE. Scribble mutants cooperate with oncogenic ras or notch to cause neoplastic overgrowth in drosophila. EMBO J (2003) 22:5769–79. doi: 10.1093/emboj/cdg548 PMC275405 14592975

[7] Pagliarini RA Xu T. A genetic screen in drosophila for metastatic behavior. Science (2003) 302:1227–31. doi: 10.1126/science.1088474 14551319

[8] Igaki T Pagliarini RA Xu T. Loss of cell polarity drives tumor growth and invasion through JNK activation in drosophila. Curr Biol (2006) 16:1139–46. doi: 10.1016/j.cub.2006.04.042 16753569

[9] Perez E Lindblad JL Bergmann A. Tumor-promoting function of apoptotic caspases by an amplification loop involving ROS, macrophages and JNK in drosophila. Elife (2017) 6:e26747. doi: 10.7554/eLife.26747 28853394 PMC5779227

[10] Zhu M Xin T Weng S Gao Y Zhang Y Li Q. Activation of JNK signaling links lgl mutations to disruption of the cell polarity and epithelial organization in drosophila imaginal discs. Cell Res (2010) 20:242–5. doi: 10.1038/cr.2010.2 20066009

[11] Ciapponi L Jackson DB Mlodzik M Bohmann D. Drosophila fos mediates ERK and JNK signals *via* distinct phosphorylation sites. Genes Dev (2001) 15:1540–53. doi: 10.1101/gad.886301 PMC312716 11410534

[12] Zeke A Misheva M Remenyi A Bogoyevitch MA. JNK signaling: regulation and functions based on complex protein-protein partnerships. Microbiol Mol Biol Rev (2016) 80:793–835. doi: 10.1128/MMBR.00043-14 27466283 PMC4981676

[13] Bennett V Baines AJ. Spectrin and ankyrin-based pathways: metazoan inventions for integrating cells into tissues. Physiol Rev (2001) 81:1353–92. doi: 10.1152/physrev.2001.81.3.1353 11427698

[14] Lee SK Thomas GH. Rac1 modulation of the apical domain is negatively regulated by beta (Heavy)-spectrin. Mech Dev (2011) 128:116–28. doi: 10.1016/j.mod.2010.11.004 21111816

[15] Fletcher GC Elbediwy A Khanal I Ribeiro PS Tapon N Thompson BJ. The spectrin cytoskeleton regulates the hippo signalling pathway. EMBO J (2015) 34:940–54. doi: 10.15252/embj.201489642 PMC4388601 25712476

[16] Baek SH Kwon YC Lee H Choe KM. Rho-family small GTPases are required for cell polarization and directional sensing in drosophila wound healing. Biochem Biophys Res Commun (2010) 394:488–92. doi: 10.1016/j.bbrc.2010.02.124 20184864

[17] Wertheimer E Gutierrez-Uzquiza A Rosemblit C Lopez-Haber C Sosa MS Kazanietz MG. Rac signaling in breast cancer: a tale of GEFs and GAPs. Cell Signal (2012) 24:353–62. doi: 10.1016/j.cellsig.2011.08.011 PMC3312797 21893191

[18] Archibald A Mihai C Macara IG McCaffrey L. Oncogenic suppression of apoptosis uncovers a Rac1/JNK proliferation pathway activated by loss of Par3. Oncogene (2015) 34:3199–206. doi: 10.1038/onc.2014.242 PMC4324374 25109337

[19] Kirkpatrick RB Matico RE McNulty DE Strickler JE Rosenberg M. An abundantly secreted glycoprotein from drosophila melanogaster is related to mammalian secretory proteins produced in rheumatoid tissues and by activated macrophages. Gene (1995) 153:147–54. doi: 10.1016/0378-1119(94)00756-I 7875581

[20] Kawamura K Shibata T Saget O Peel D Bryant PJ. A new family of growth factors produced by the fat body and active on drosophila imaginal disc cells. Development (1999) 126:211–9. doi: 10.1242/dev.126.2.211 9847235

[21] Kucerova L Kubrak OI Bengtsson JM Strnad H Nylin S Theopold U. Slowed aging during reproductive dormancy is reflected in genome-wide transcriptome changes in drosophila melanogaster. BMC Genomics (2016) 17:50. doi: 10.1186/s12864-016-2383-1 26758761 PMC4711038

[22] Pesch YY Riedel D Patil KR Loch G Behr M. Chitinases and imaginal disc growth factors organize the extracellular matrix formation at barrier tissues in insects. Sci Rep (2016) 6:18340. doi: 10.1038/srep18340 26838602 PMC4738247

[23] Yadav S Eleftherianos I. The imaginal disc growth factors 2 and 3 participate in the drosophila response to nematode infection. Parasite Immunol (2018) 40:e12581. doi: 10.1111/pim.12581 30107045

[24] Krautz R Khalili D Theopold U. Tissue-autonomous immune response regulates stress signalling during hypertrophy. Elife (2020) 9:e64919. doi: 10.7554/eLife.64919 33377870 PMC7880693

[25] Khalili D Kalcher C Baumgartner S Theopold U. Anti-fibrotic activity of an antimicrobial peptide in a drosophila model. J Innate Immun (2021) 13:376–90. doi: 10.1159/000516104 PMC8613551 34000729

[26] Hauptmann G Söll I Krautz R Theopold U. Multi-target Chromogenic Whole-mount In Situ Hybridization for Comparing Gene Expression Domains in Drosophila Embryos. J Vis Exp (2016) 107:e53830. doi: 10.3791/53830 PMC4781704 26862978

[27] Morera E Steinhauser SS Budkova Z Ingthorsson S Kricker J Krueger A. YKL-40/CHI3L1 facilitates migration and invasion in HER2 overexpressing breast epithelial progenitor cells and generates a niche for capillary-like network formation. In Vitro Cell Dev Biol Anim (2019) 55:838–53. doi: 10.1007/s11626-019-00403-x PMC6881255 31482369

[28] Karlsson C Korayem AM Scherfer C Loseva O Dushay MS Theopold U. Proteomic analysis of the drosophila larval hemolymph clot. J Biol Chem (2004) 279:52033–41. doi: 10.1074/jbc.M408220200 15466469

[29] Palmerini V Monzani S Laurichesse Q Loudhaief R Mari S Cecatiello V. Drosophila TNFRs grindelwald and wengen bind eiger with different affinities and promote distinct cellular functions. Nat Commun (2021) 12:2070. doi: 10.1038/s41467-021-22080-9 33824334 PMC8024323

[30] Diwanji N Bergmann A. The beneficial role of extracellular reactive oxygen species in apoptosis-induced compensatory proliferation. Fly (Austin) (2017) 11:46–52. doi: 10.1080/19336934.2016.1222997 27575697 PMC5354222

[31] Chatterjee N Bohmann D. A versatile PhiC31 based reporter system for measuring AP-1 and Nrf2 signaling in drosophila and in tissue culture. PloS One (2012) 7:e34063. doi: 10.1371/journal.pone.0034063 22509270 PMC3324472

[32] Uhlirova M Bohmann D. JNK- and fos-regulated Mmp1 expression cooperates with ras to induce invasive tumors in drosophila. EMBO J (2006) 25:5294–304. doi: 10.1038/sj.emboj.7601401 PMC1636619 17082773

[33] La Marca JE Richardson HE. Two-faced: roles of JNK signalling during tumourigenesis in the drosophila model. Front Cell Dev Biol (2020) 8:42. doi: 10.3389/fcell.2020.00042 32117973 PMC7012784

[34] Bilder D Ong K Hsi TC Adiga K Kim J. Tumour-host interactions through the lens of drosophila. Nat Rev Cancer (2021) 21:687–700. doi: 10.1038/s41568-021-00387-5 34389815 PMC8669834

[35] Tran DT Kelly G. Ten Hagen Real-time insights into regulated exocytosis. J Cell Sci (2017) 130 (8):1355–1363. doi: 10.1242/jcs.193425 PMC5399783 28302911

[36] Bartlett BJ Isakson P Lewerenz J Sanchez H Kotzebue RW Cumming RC. p62, Ref(2)P and ubiquitinated proteins are conserved markers of neuronal aging, aggregate formation and progressive autophagic defects. Autophagy (2011) 7:572–83. doi: 10.4161/auto.7.6.14943 PMC3127048 21325881

[37] Asano K Miwa M Miwa K Hanayama R Nagase H Nagata S. Masking of phosphatidylserine inhibits apoptotic cell engulfment and induces autoantibody production in mice. J Exp Med (2004) 200 (4):459–467. doi: 10.1084/jem.20040342 PMC2211927 15302904

[38] Recouvreux MV Commisso C. Macropinocytosis: a metabolic adaptation to nutrient stress in cancer. Front Endocrinol (Lausanne) (2017) 8:261. doi: 10.3389/fendo.2017.00261 29085336 PMC5649207

[39] Williams ST Smith AN Cianci CD Morrow JS Brown TL. Identification of the primary caspase 3 cleavage site in alpha II-spectrin during apoptosis. Apoptosis (2003) 8:353–61. doi: 10.1023/A:1024168901003 12815278

[40] Zhao T Su Z Li Y Zhang X You Q. Chitinase-3 like-protein-1 function and its role in diseases. Signal Transduct Target Ther (2020) 5:201. doi: 10.1038/s41392-020-00303-7 32929074 PMC7490424

[41] Kucerova L Broz V Arefin B Maaroufi HO Hurychova J Strnad H. The drosophila chitinase-like protein IDGF3 is involved in protection against nematodes and in wound healing. J Innate Immun (2015) 8:199–210. doi: 10.1159/000442351 PMC6738885 26694862

[42] Lee CG Da Silva CA Dela Cruz CS Ahangari F Ma B Kang MJ. Role of chitin and chitinase/chitinase-like proteins in inflammation, tissue remodeling, and injury. Annu Rev Physiol (2011) 73:479–501. doi: 10.1146/annurev-physiol-012110-142250 21054166 PMC3864643

[43] Fogarty CE Bergmann A. Killers creating new life: caspases drive apoptosis-induced proliferation in tissue repair and disease. Cell Death Differ (2017) 24:1390–400. doi: 10.1038/cdd.2017.47 PMC5520457 28362431

[44] Yeh YT Hou MF Chung YF Chen YJ Yang SF Chen DC. Decreased expression of phosphorylated JNK in breast infiltrating ductal carcinoma is associated with a better overall survival. Int J Cancer (2006) 118:2678–84. doi: 10.1002/ijc.21707 16381010

[45] Tang H Sun Y Shi Z Huang H Fang Z Chen J. YKL-40 induces IL-8 expression from bronchial epithelium *via* MAPK (JNK and ERK) and NF-kappaB pathways, causing bronchial smooth muscle proliferation and migration. J Immunol (2013) 190:438–46. doi: 10.4049/jimmunol.1201827 23197259

[46] Insua-Rodriguez J Pein M Hongu T Meier J Descot A Lowy CM. Stress signaling in breast cancer cells induces matrix components that promote chemoresistant metastasis. EMBO Mol Med (2018) 10:e9003. doi: 10.15252/emmm.201809003 30190333 PMC6180299

[47] Juliano RL Kimelberg HK Papahadjopoulos D. Synergistic effects of a membrane protein (spectrin) and Ca 2+ on the Na + permeability of phospholipid vesicles. Biochim Biophys Acta (1971) 241:894–905. doi: 10.1016/0005-2736(71)90017-4 5003695

[48] Pinder JC Bray D Gratzer WB. Actin polymerisation induced by spectrin. Nature (1975) 258:765–6. doi: 10.1038/258765a0 1207764

[49] Hardy B Schrier SL. The role of spectrin in erythrocyte ghost endocytosis. Biochem Biophys Res Commun (1978) 81:1153–61. doi: 10.1016/0006-291X(78)91257-3 96830

[50] Lattner J Leng W Knust E Brankatschk M Flores-Benitez D. Crumbs organizes the transport machinery by regulating apical levels of PI(4,5)P2 in drosophila. Elife (2019) 8:e50900. doi: 10.7554/eLife.50900 31697234 PMC6881148

[51] Ackermann A Brieger A. The role of nonerythroid spectrin alphaII in cancer. J Oncol (2019) 2019:7079604. doi: 10.1155/2019/7079604 31186638 PMC6521328

[52] Yang P Yang Y Sun P Tian Y Gao F Wang C. betaII spectrin (SPTBN1): biological function and clinical potential in cancer and other diseases. Int J Biol Sci (2021) 17:32–49. doi: 10.7150/ijbs.52375 33390831 PMC7757025

[53] Ziemnicka-Kotula D Xu J Gu H Potempska A Kim KS Jenkins EC. Identification of a candidate human spectrin src homology 3 domain-binding protein suggests a general mechanism of association of tyrosine kinases with the spectrin-based membrane skeleton. J Biol Chem (1998) 273:13681–92. doi: 10.1074/jbc.273.22.13681 9593709

[54] Macoska JA Xu J Ziemnicka D Schwab TS Rubin MA Kotula L. Loss of expression of human spectrin src homology domain binding protein 1 is associated with 10p loss in human prostatic adenocarcinoma. Neoplasia (2001) 3:99–104. doi: 10.1038/sj.neo.7900145 11420744 PMC1505418

[55] Dubielecka PM Cui P Xiong X Hossain S Heck S Angelov L. Differential regulation of macropinocytosis by Abi1/Hssh3bp1 isoforms. PloS One (2010) 5:e10430. doi: 10.1371/journal.pone.0010430 20479892 PMC2866655

[56] Ritter M Bresgen N Kerschbaum HH. From pinocytosis to methuosis-fluid consumption as a risk factor for cell death. Front Cell Dev Biol (2021) 9:651982. doi: 10.3389/fcell.2021.651982 34249909 PMC8261248

[57] Maxson ME Sarantis H Volchuk A Brumell JH Grinstein S. Rab5 regulates macropinocytosis by recruiting the inositol 5-phosphatases OCRL and Inpp5b that hydrolyse PtdIns(4,5)P2. J Cell Sci (2021) 134(7):jcs252411. doi: 10.1242/jcs.252411 33722976

[58] Shubin AV Demidyuk IV Komissarov AA Rafieva LM Kostrov SV. Cytoplasmic vacuolization in cell death and survival. Oncotarget (2016) 7:55863–89. doi: 10.18632/oncotarget.10150 PMC5342458 27331412

[59] Yan G Dawood M Bockers M Klauck SM Fottner C Weber MM. Multiple modes of cell death in neuroendocrine tumors induced by artesunate. Phytomedicine (2020) 79:153332. doi: 10.1016/j.phymed.2020.153332 32957040

[60] Mehrotra S Maqbool SB Kolpakas A Murnen K Calvi BR. Endocycling cells do not apoptose in response to DNA rereplication genotoxic stress. Genes Dev (2008) 22:3158–71. doi: 10.1101/gad.1710208 PMC2593612 19056894

[61] Zhang B Mehrotra S Ng WL Calvi BR. Low levels of p53 protein and chromatin silencing of p53 target genes repress apoptosis in drosophila endocycling cells. PloS Genet (2014) 10:e1004581. doi: 10.1371/journal.pgen.1004581 25211335 PMC4161308

[62] Araki M Kurihara M Kinoshita S Awane R Sato T Ohkawa Y. Anti-tumour effects of antimicrobial peptides, components of the innate immune system, against haematopoietic tumours in drosophila mxc mutants. Dis Model Mech (2019) 12(6):dmm037721. doi: 10.1242/dmm.037721 31160313 PMC6602314

[63] Parvy JP Yu Y Dostalova A Kondo S Kurjan A Bulet P. The antimicrobial peptide defensin cooperates with tumour necrosis factor to drive tumour cell death in drosophila. Elife (2019) 8:e45061. doi: 10.7554/eLife.45061 31358113 PMC6667213

[64] Kzhyshkowska J Larionova I Liu T. YKL-39 as a potential new target for anti-angiogenic therapy in cancer. Front Immunol (2019) 10:2930. doi: 10.3389/fimmu.2019.02930 32038607 PMC6988383

