# Supplementary material for: Chitinase-like proteins promoting tumorigenesis through disruption of cell polarity via enlarged endosomal vesicles
Source: Front Oncol. 2023 Apr 28;13:1170122. doi: 10.3389/fonc.2023.1170122 (PMC10175591; doi:10.3389/fonc.2023.1170122)
Supplement: Supplementary file 1 [file DataSheet_1.pdf]

Supplementary Material

Chitinase-like proteins promote tumorigenesis through disruption of cell polarity and formation of enlarged vesicles

Dilan Khalili[1], Martin Kunc[1], Sarah Herbrich[1], Anna M. Müller[1], Ulrich Theopold[1*]

*** Correspondence:** Ulrich Theopold: uli.theopold@su.se

1 Supplementary figures

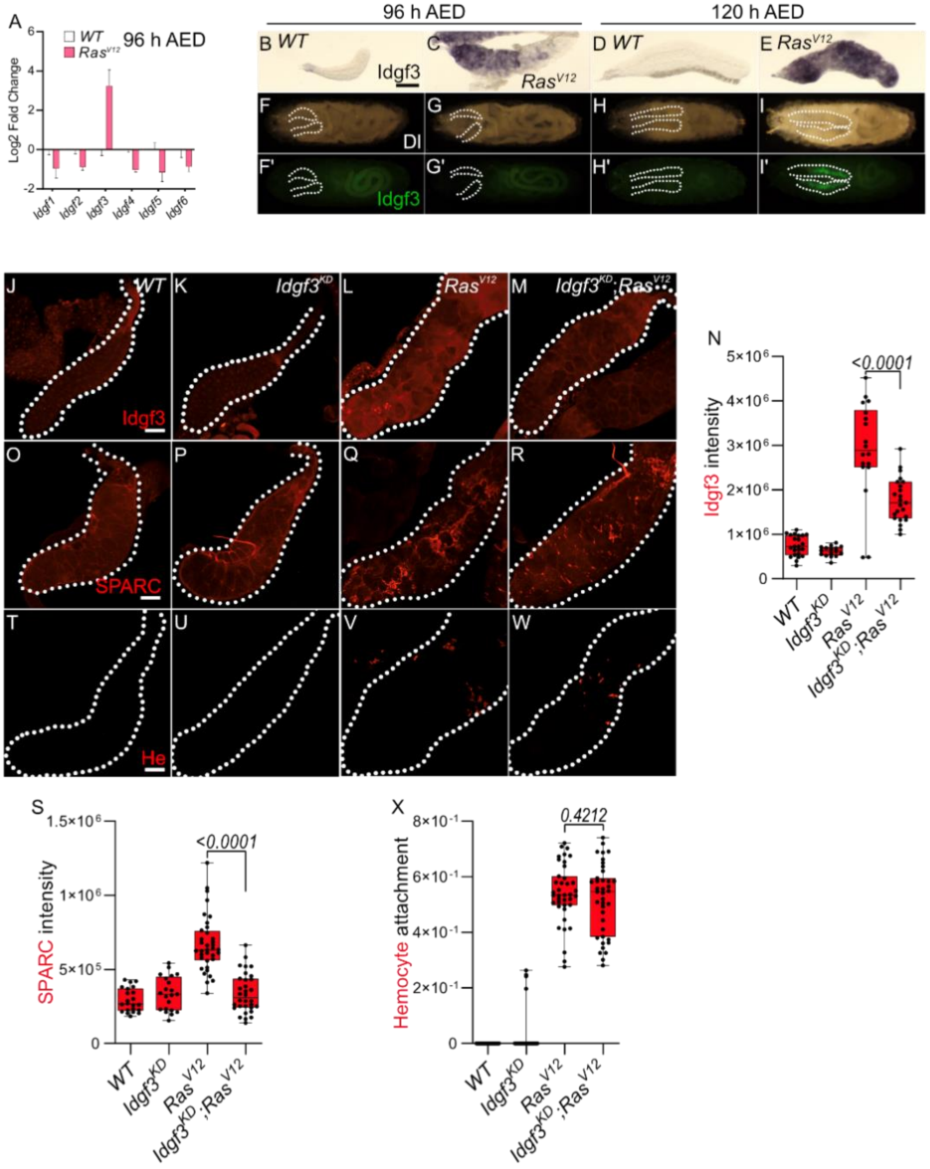

Figure S1

Idgf3 characterization and tumor effects

(A) qPCR data showing induction of *Idgf3* in 96 h AED *Ras*^{V12} glands. **(B-E)** ISH showing Idgf3 distribution throughout the SG at 96 h and 120 h AED. **(F-I')** Whole larvae images showing Idgf3::GFP localization. **(J-M)** Antibody staining against Idgf3 showing efficiency of *Idgf3*^{KD}. **(N)** Intensity quantification showing *Idgf3*^{KD} reduction efficiency of Idgf3. **(O-R)** SPARC staining displaying reduced fibrosis in *Idgf3*^{KD}*;Ras*^{V12} SG. **(S)** Quantification showing reduced SPARC intensity in *Idgf3*^{KD}*;Ras*^{V12} SG. **(T-W)** Hemocytes staining. **(X)** Quantification showing no effect on attached hemocytes in *Idgf3*^{KD}*;Ras*^{V12} SG. Data in **(A)** represent 3 independent replicas summarized as mean ± SD Scale bars in **(B-E)** represent 0.3 mm and **(J-W)** 100 µm. Boxplot in **(N, S and X)** represent at least 20 SG pairs. Whisker length min to max, bar represent median. P-value quantified with Student's t-test.

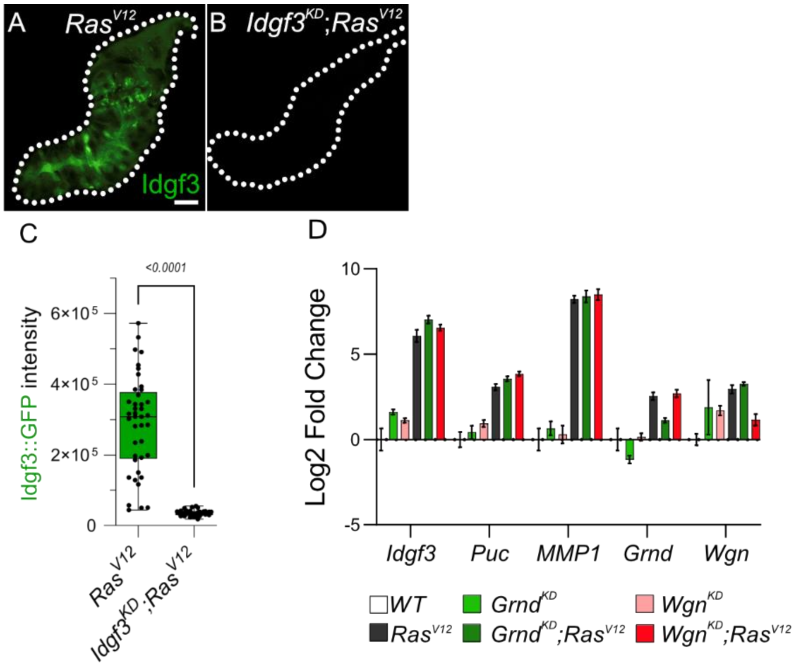

Figure S2

Non-canonical JNK regulation and activation in *Ras*^{V12} glands

(A-B) *Idgf3*^{KD}*;Ras*^{V12} showing reduced Idgf3::GFP intensity quantified in **(C)**. **(D)** qPCR showing *Grnd*^{KD} and *Wgn*^{KD} are downregulating respective mRNA efficiently in *Ras*^{V12} background. Scale bars in **(A-B)** represent 100 µm. Boxplot in **(C)** represent at least 20 SG pairs. Whisker length min to max, bar represent median. Data in **(D)** represent 4 independent replicas summarized as mean ± SD. P-value quantified with Student's t-test.

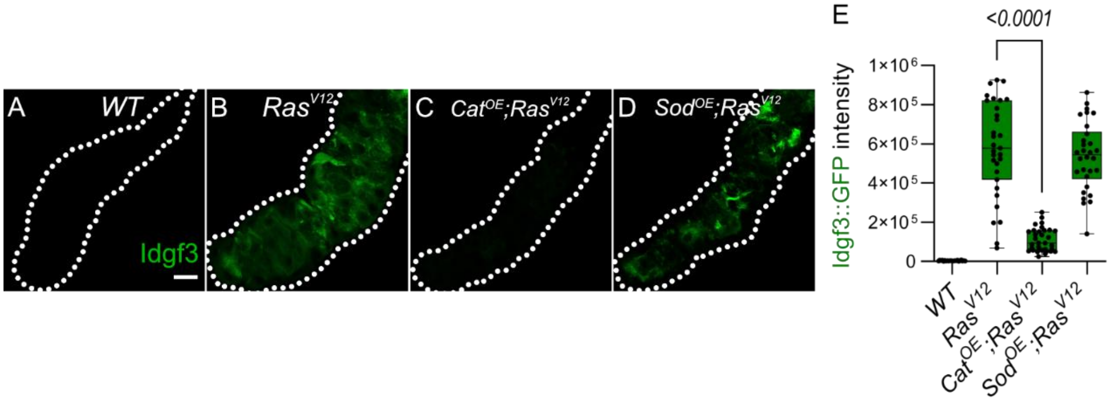

Figure S3
Idgf3 and JNK regulation by ROS
(A-D) Effect on Idgf3::GFP by reductases. **(E)** Quantification showing reduced Idgf3::GFP intensity in *Cat*^{OE}*;Ras*^{V12}. Scale bars in **(A-D)** represent 100 µm. Boxplot in **(E)** represent at least 20 SG pairs. Whisker length min to max, bar represent median. P-value quantified with Student's t-test.

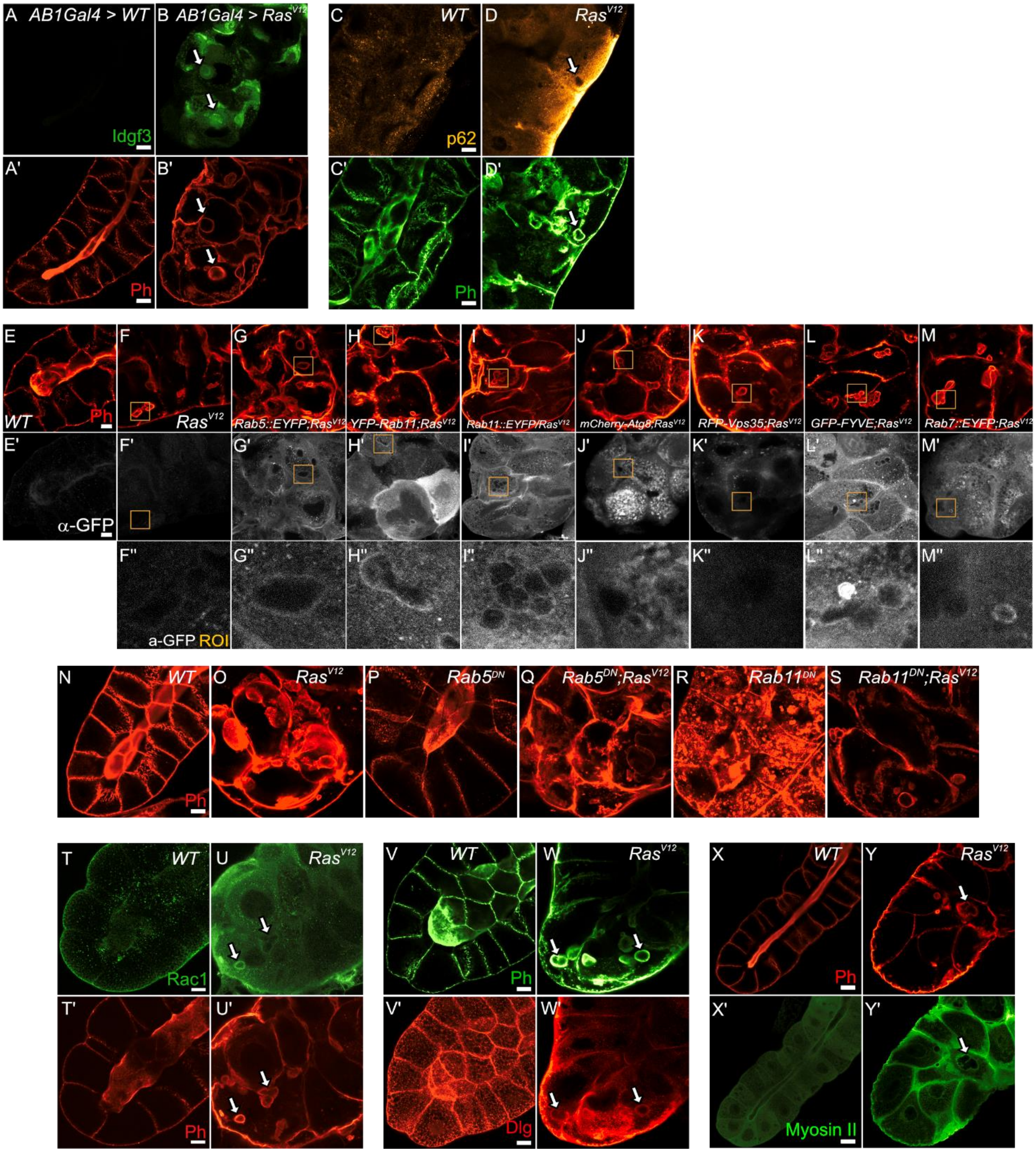

Figure S4

Enlarged vesicles (EnVs) characterization

(A-B') Idgf3 induction and accumulation are independent of the SG driver. **(C-D')** p62 staining showing no accumulation in EnVs. **(E-M'')** Vesicle markers showing EnVs stained for RAb5, Rab11 and FYVE. **(N-S)** Phalloidin staining showing no effect on the formation of EnVs in *Rab5*^{DN}*;Ras*^{V12} and *Rab11*^{DN}*;Ras*^{V12} glands. **(T-U')** Rac1 staining showing EnVs co-localizing with Phalloidin. **(V-W')** Phalloidin and Dlg staining showing to co-localize at EnVs. **(X-Y')** Phalloidin and GFP tagged Myosin II showing to co-localize at EnVs. Scale bars in **(A-Y')** represent 20 μm.

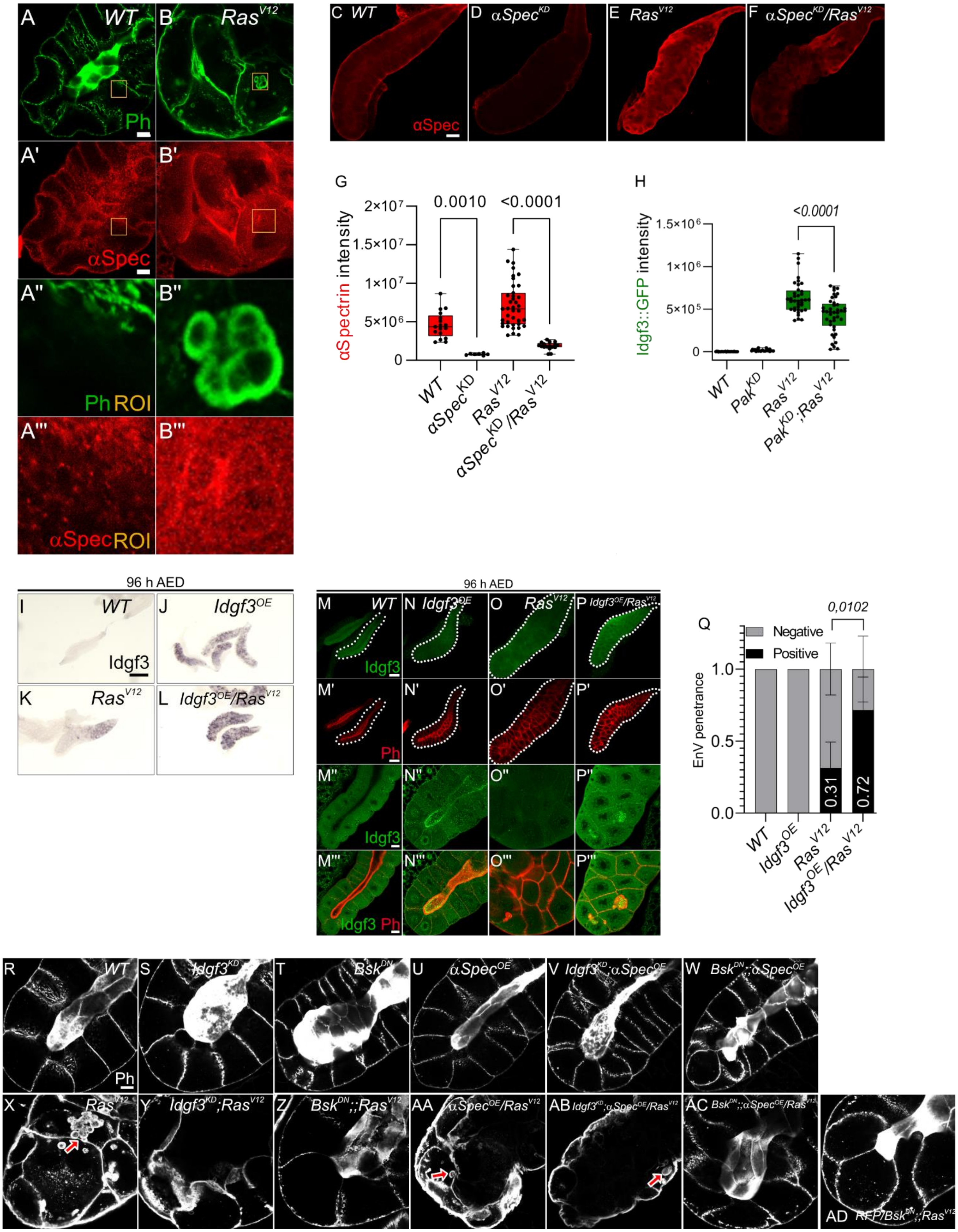
A WT
B RasV12
Ph
A' B'
αSpec
A'' B''
Ph ROI
A''' B'''
αSpec ROI
C WT
D αSpecKD
E RasV12
F αSpecKD/RasV12
αSpec
G
αSpectrin intensity
2×10⁷
1.5×10⁷
1×10⁷
5×10⁶
0
0.0010
<0.0001
WT
αSpecKD
RasV12
αSpecKD/RasV12
H
Idgf3::GFP intensity
1.5×10⁶
1×10⁶
5×10⁵
0
<0.0001
WT
PakKD
RasV12
PakKD;RasV12
96 h AED
I WT
J Idgf3OE
Idgf3
K RasV12
L Idgf3OE/RasV12
96 h AED
M WT
N Idgf3OE
O RasV12
P Idgf3OE/RasV12
Idgf3
M' N' O' P'
Ph
M'' N'' O'' P''
Idgf3
M''' N''' O''' P'''
Idgf3 Ph
Q
0,0102
Negative
Positive
EnV penetrance
1.0
0.5
0.0
0.31
0.72
WT
Idgf3OE
RasV12
Idgf3OE/RasV12
R WT
S Idgf3KD
T BskDN
U αSpecOE
V Idgf3KD;αSpecOE
W BskDN;;αSpecOE
Ph
X RasV12
Y Idgf3KD;RasV12
Z BskDN;;RasV12
AA αSpecOE/RasV12
AB Idgf3KD;αSpecOE/RasV12
AC BskDN;;αSpecOE/RasV12
AD RFP/BskDN;;RasV12

Figure S5

(A-B'') Phalloidin and αSpectrin staining shows colocalization at EnVs. **(C-F)** αSpectrin staining showing reduction in αSpectrin^{KD} quantified in **(G)**. **(H)** Quantification showing increased level of Idgf3::GFP, no formation of EnVs and Idgf3 in *Pak^{KD};Ras^{V12}* glands. **(I-L)** ISH showing Idgf3 distribution in Idgf3^{OE}/Ras^{V12} glands. **(M-P''')** Idgf3 staining EnVs in Idgf3^{OE};Ras^{V12} glands. **(Q)** EnV penetrance quantification showing increased amount of glands with EnVs in Idgf3^{OE};Ras^{V12}. **(R-AD)** Epistatic analysis between Idgf3 and αSpectrin. Phalloidin staining showing EnVs presence in *Idgf3^{KD};αSpec^{OE}/ Ras^{V12}*. Scale bars in **(A-B', M''-P''', R-AD)** represent 20 µm, (C-F, M-P') represents 100 µm, **(I-L)** represents 0.3 mm. Boxplot in **(G-H)** represent at least 20 SG pairs. Bar plot in **(Q)** represent 3 independent replicas with at least 10 SGs pairs summarized as relative value ± SD. Whisker length min to max, bar represent median. P-value quantified with Student's t-test.

2 Supplementary tables

Supplementary table 1.

REAGENT or RESOURCE	SOURCE	IDENTIFIER
**Antibodies**		
anti-IDGF3 (rabbit)	Kucerova et al., 2016	Idgf3
anti-SPARC	Khalili et al., 2021	Sparc
anti-Hemese (mouse monoclonal)	István Andó	H2
pJNK	Cell Signaling Technology	9255
Rac1 fitc conjugated mouse	BD Bioscience Pharmingen	610652
αSpectrin	DSHB	3A9
anti-GFP (mouse monoclonal)	ThemoFisher Scientific	A11120
anti-mouse-IgG Alexa546 (goat polyclonal)	ThemoFisher Scientific	A11030
anti-rabbit-IgG Alexa568 (goat polyclonal)	ThemoFisher Scientific	A21069
anti-mouse-IgG, Alexa Fluor 488	ThemoFisher Scientific	A11001
anti-rabbit-IgG, Alexa Fluor 488	ThemoFisher Scientific	A11008
**Chemicals, peptides, and recombinant proteins**		
DAPI	Sigma-Aldrich	D9542
Alexa Fluor 488 Phalloidin	ThermoFisher Scientific	A12379
Alexa Fluor 546 Phalloidin	ThermoFisher Scientific	A22283
Vancomycin	Sigma-Aldrich	V2002
Neomycin	Sigma-Aldrich	N1876
Metronidazole	Sigma-Aldrich	M3761
Carbenicillin	Sigma-Aldrich	C1389
**Critical commercial assays**		
RNAquesous Micro Kit	ThemoFisher Scientific	AM1931
**Experimental models: organisms/strains**		
D. melanogaster w^{1118}		
D. melanogaster Bx^{ms1096}	BDSC	8860
D. melanogaster UAS-Ras^{V12}	BDSC	4847
D. melanogaster Idgf3::GFP	Kucerova et al., 2016	

D. melanogaster UAS-Idgf3^{KD}	BDSC	
D. melanogaster UAS-Grnd^{KD}	VDRC	104538
D. melanogaster UAS-Wgn^{KD}	BDSC	55275
D. melanogaster UAS-Bsk^{KD}	BDSC	36643
D. melanogaster UAS-Bsk^{KD}	VDRC	104569
D. melanogaster UAS-Drs	B. Lemaitre	
D. melanogaster UAS-IRC	WJL	
D. melanogaster UAS- CatA	BDSC	24621
D. melanogaster UAS-SodA	BDSC	33605
D. melanogaster UAS-MFG-E8	Dr Nakanishi	
D. melanogaster UAS-MFG-E8ΔC	Dr Nakanishi	
D. melanogaster Rab5::EYFP	BDSC	62543
D. melanogaster UAS-YFP.Rab11	BDSC	50782
D. melanogaster Rab7::EYFP	BDSC	62545
D. melanogaster Rab11::EYFP	BDSC	62549
D. melanogaster UAS-mCherry-Atg8	BDSC	37750
D. melanogaster RFP-Vps35	BDSC	66527
D. melanogaster GFP-FYVE	BDSC	42712
D. melanogaster UAS-YFP.Rab5^{DN}	BDSC	9771
D. melanogaster UAS-YFP.Rab11^{DN}	BDSC	9792
D. melanogaster UAS-Rac1	BDSC	6293
D. melanogaster UAS-Pak^{KD}	BDSC	41714
D. melanogaster UAS-βSpect^{KD}	BDSC	
D. melanogaster UAS-αSpec^{KD}	BDSC	31209
D. melanogaster UAS-Idgf3	Kucerova et al., 2016<br>BDSC	52658
D. melanogaster mCD8::RFP	Krautz et al., 2020<br>BDSC	27400
D. melanogaster αSpec.UPS	BDSC	32005
D. melanogaster TRE.GFP	D. Bohmann	
D. melanogaster hs-Flp^{122}*; UAS-mCherry/CyO, Act-GFP*^{JMR1}*; UAS-Flp, Act5C > CD2 > Gal4/TM6b*	Duan et al., 2020	

Oligonucleotides

Drosophila Idgf1 forward: 5’- CTCAGTGTGACAAGTTTGACCC-3’	
Drosophila Idgf1 reverse: 5’- CCTTGGCGCAAATACGAGTTG-3’	
Drosophila Idgf2 forward: 5’-CAAGAAAGGTTCACGGTGATCT-3’	
Drosophila Idgf2 reverse: 5’-AACTGCTCCTTATGAAGGGCT-3’	
Drosophila Idgf3 forward: 5’-GTGCCGCTCTGAAACAAAAT-3’	
Drosophila Idgf3 reverse: 5’-ACGGGGCATCATAGTACCAG-3’	
Drosophila Idgf4 forward: 5’-TTACTATGACGGCAACAGTTTTGT-3’	
Drosophila Idgf4 reverse: 5’-GGCATATCCGTAGACCAGATG-3’	
Drosophila Idgf5 forward: 5’-CAGAGGTTGGAAAACTGGTGT-3’	
Drosophila Idgf5 reverse: 5’-GTTCAGCCAGCGACATTTGG-3’	
Drosophila Idgf6 forward: 5’-ACCGCTCTACTCCGTGATGT-3’	
Drosophila Idgf6 reverse: 5’-GCGGGAATGTCGTAGAACAG-3’	
Drosophila Puc forward: 5’-CGTCATCATCAACGGCAAT-3’	
Drosophila Puc reverse: 5’-AGGCGGGGTGTGTTTCTAT-3’	
Drosophila MMP1 forward: 5’-GTTTCCACCACCACACAGG-3’	
Drosophila MMP1 reverse: 5’-GCAGAGGCGGGTAGATAGC-3’	
Drosophila Grnd forward: 5’-CACAACTACGATGCGTTTCTGT-3’	PP32778
Drosophila Grnd reverse: 5’-CATCTCGGCTTTTAACGGCTC-3’	PP32778

Drosophila Wng forward: 5’-ACCATCTGCGGTTCCATATACG-3’		PP26269
Drosophila Wgn reverse: 5’-GTGCTCATACTCGGAGGACTT-3’		PP26269
Drosophila Hid forward: 5’-TCTACGAGTGGGTCAGGATGT-3’		
Drosophila Hid reverse: 5’-GCGGATACTGGAAGATTTGC-3’		
Drosophila Rac1 forward: 5’-GGAAATCGAACCATGCAGGC-3’		PD70033
Drosophila Rac1 reverse: 5’-GTCGAACACGGTGGGTATGT-3’		PD70033
Drosophila αSpec forward: 5’-CGACCGCCCCTATGTAACTA-3’		PD70352
Drosophila αSpec reverse: 5’-CACGCAGTAGTCAGCCATGT-3’		PD70352
Drosophila Karst forward: 5’-GGACAACCTTAACCATGCCTT-3’		PP36727
Drosophila Karst reverse: 5’-AGTAGGAGGCCACATAGGTCA-3’		PP36727
Human CH3L1 forward: 5’-TCAAGAACAGGAACCCCAAC-3’		
Human CH3L1 reverse: 5’-AAATTCGGCCTTCATTTCCT -3’		
Human CH3L1 forward: 5’-CAGGGAGGCAAATGATTGAT -3’		
Human CH3L1 reverse: 5’-CCACCTTCTCTGATGGCATT -3’		
Drosophila Rpl32 forward: 5’-CGGATCGATATGCTA-3’		
Drosophila Rpl32 reverse: 5’-CGACGCACTCTGTTG-3’		
**Recombinant DNA**		
Idgf3 cDNA	Drosophila Genomics Resource Center (DGRC)	GH07453
**Software and algorithms**		
FIJI (ImageJ)	https://imagej.net/Fiji/Downloads	Version 1.53j
GraphPad Prism	https://www.graphpad.com/	Version 9.1.2
Affinity Designer	https://affinity.serif.com/en-gb/	Version 1.9.2.1035
Zen blue	https://www.zeiss.com/	Version 2.3
AxioVision LE	https://www.zeiss.com/	Version 4.8.2.0

2.1 Cross list:

Fig. 1

A:

♀ *w^{1118},Beadex-Gal4 (Bx)* > ♂*w^{1118}*

♀*Bx* > ♂ *w^{1118};;UAS-Ras^{V12}* (*Ras^{V12}*)

B-C:

♀ *Bx; Idgf3::GFP* > ♂*w^{1118}*

♀ *Bx; Idgf3::GFP* > ♂ *Ras^{V12}*

D-Q:
♀*Bx* > ♂w^{1118}
♀*Bx* > ♂ Ras^{V12}
♀*Bx* > ♂ w^{1118}; $Idgf3^{KD}$
♀*Bx* > ♂ w^{1118}; $Idgf3^{KD}$; Ras^{V12}

Fig. S1
A:
♀*Bx* > ♂w^{1118}
♀*Bx* > ♂ Ras^{V12}

B-E:
♀*Bx* > ♂w^{1118}
♀*Bx* > ♂ Ras^{V12}

F-I':
♀ *Bx; Idgf3::GFP* > ♂w^{1118}
♀ *Bx; Idgf3::GFP* > ♂ Ras^{V12}

J-X:
♀*Bx* > ♂w^{1118}
♀*Bx* > ♂ w^{1118}; $Idgf3^{KD}$
♀*Bx* > ♂ Ras^{V12}
♀*Bx* > ♂ w^{1118}; $Idgf3^{KD}$; Ras^{V12}

Fig. 2
A-E:
♀ *Bx; Idgf3::GFP* > ♂ Ras^{V12}
♀ *Bx; Idgf3::GFP* > ♂ w^{1118}; $Grnd^{KD}$; Ras^{V12}
♀ *Bx; Idgf3::GFP* > ♂ w^{1118}; Wgn^{KD}; Ras^{V12}
♀ *Bx; Idgf3::GFP* > ♂ w^{1118}; $JNK^{KD\ [36643/Bl]}$; Ras^{V12}

Fig. S2
A-C:
♀ *Bx; Idgf3::GFP* > ♂ Ras^{V12}
♀ *Bx; Idgf3::GFP* > ♂ w^{1118}; $Idgf3^{KD}$; Ras^{V12}

D:
♀*Bx* > ♂w^{1118}
♀ *Bx* > ♂ Ras^{V12}
♀ *Bx* > ♂ w^{1118}; $Grnd^{KD}$
♀ *Bx* > ♂ w^{1118}; Wgn^{KD}
♀ *Bx* > ♂ w^{1118}; $Grnd^{KD}$; Ras^{V12}
♀ *Bx* > ♂ w^{1118}; Wgn^{KD}; Ras^{V12}

Fig. 3
A-I:
♀ *Bx* > ♂ w^{1118}; *Idgf3::GFP*

♀ *Bx;; IRC-OE* > ♂ *w^{1118}; Idgf3::GFP*
♀ *Bx* > ♂ *w^{1118}; Idgf3::GFP; Ras^{V12}*
♀ *Bx;; IRC-OE* > ♂ *w^{1118}; Idgf3::GFP; Ras^{V12}*

J-O:
♀ *Bx* > ♂ *w^{1118}*
♀ *Bx;; IRC-OE* > ♂ *w^{1118}*
♀ *Bx* > ♂ *Ras^{V12}*
♀ *Bx;; IRC-OE* > ♂ *Ras^{V12}*

P-T:
♀ *Bx* > ♂ *w^{1118}*; TRE.GFP/ CyO.GFP
♀ *Bx;; IRC-OE* > ♂ *w^{1118}*; TRE.GFP/ CyO.GFP
♀ *Bx* > ♂ *w^{1118}*; TRE.GFP/ CyO.GFP; *Ras^{V12}*
♀ *Bx;; IRC-OE* > ♂ *w^{1118}*; TRE.GFP/ CyO.GFP; *Ras^{V12}*

Fig. S3
A-E:
♀ *Bx; Idgf3::GFP* > ♂ *w^{1118}*
♀ *Bx; Idgf3::GFP* > ♂ *w^{1118}; Ras^{V12}*
♀ *Bx; Idgf3::GFP* > ♂ *w^{1118}; Cat-OE; Ras^{V12}*
♀ *Bx; Idgf3::GFP* > ♂ *w^{1118}; SodA-OE; Ras^{V12}*

Fig. 4
B-C':
♀*Bx; Idgf3::GFP*> ♂ *w^{1118}*
♀*Bx; Idgf3::GFP*> ♂ *Ras^{V12}*

D:
♀*Bx* > ♂ *w^{1118}*
♀*Bx* > ♂ *Ras^{V12}*

E-J':
♀ *Bx* > ♂ *w^{1118}*
♀ *Bx* > ♂ *w^{1118}; MFG-E8*ΔC*::GFP-OE*
♀ *Bx* > ♂ *w^{1118}; MFG-E8::GFP-OE*
♀ *Bx* > ♂ *Ras^{V12}*
♀ *Bx* > ♂ *w^{1118}; MFG-E8*ΔC*::GFP-OE; Ras^{V12}*
♀ *Bx* > ♂ *w^{1118}; MFG-E8::GFP-OE; Ras^{V12}*

K-M:
♀ *Bx* > ♂ *w^{1118}*
♀ *Bx* > ♂ *Ras^{V12}*
♀ *Bx* > ♂ *w^{1118}; Pak^{CA}*

K'-M'':
♀ *Bx; Idgf3::GFP* > ♂ *w^{1118}*

♀ *Bx; Idgf3::GFP* > ♂ *Ras*^{V12}
♀ *Bx; Idgf3::GFP* > ♂ *w*^{1118}*; Pak*^{CA}

N-S:
♀ *Bx; Idgf3::GFP* > ♂ *w*^{1118}
♀ *Bx; Idgf3::GFP* > ♂ *w*^{1118}*; Pak*^{KD}
♀ *Bx; Idgf3::GFP* > ♂ *w*^{1118}*; Rac1-OE*
♀ *Bx; Idgf3::GFP* > ♂ *Ras*^{V12}
♀ *Bx; Idgf3::GFP* > ♂ *w*^{1118}*; Pak*^{KD}*; Ras*^{V12}

Fig. S4

A-B':
♀ *w*^{1118}*; AB1-Gal4*> ♂*w*^{1118}*; Idgf3::GFP*
♀ *w*^{1118}*; AB1-Gal4*> ♂*w*^{1118}*; Idgf3::GFP; Ras*^{V12}

C-D':
♀*Bx* > ♂*w*^{1118}
♀*Bx* > ♂ *Ras*^{V12}

E-M'':
♀ *Bx* > ♂ *w*^{1118}
♀ *Bx* > ♂ *Ras*^{V12}
♀ *Bx* > ♂ *w*^{1118}*; Rab5::EYFP; Ras*^{V12}
♀ *Bx* > ♂ *w*^{1118}*; YFP-Rab11; Ras*^{V12}
♀ *Bx;; Rab11::EYFP* > ♂ *Ras*^{V12}
♀ *Bx* > ♂ *w*^{1118}*; GFP-FYVE; Ras*^{V12}
♀ *Bx;; Rab7::EYFP* > ♂ *w*^{1118}*;; Ras*^{V12}

N-S:
♀ *Bx* > ♂ *w*^{1118}
♀ *Bx* > ♂ *Ras*^{V12}
♀ *Bx* > ♂ *w*^{1118}*; Rab5*^{DN}
♀ *Bx* > ♂ *w*^{1118}*; Rab5*^{DN}*; Ras*^{V12}
♀ *Bx* > ♂ *w*^{1118}*; Rab11*^{DN}
♀ *Bx* > ♂ *w*^{1118}*; Rab11*^{DN}*; Ras*^{V12}

T-U':
♀ *Bx* > ♂ *w*^{1118}
♀ *Bx* > ♂ *Ras*^{V12}

V-W':
♀ *Bx* > ♂ *w*^{1118}
♀ *Bx* > ♂ *Ras*^{V12}

X-Y':
♀ *Bx;; sqh-GFP* > ♂ *w*^{1118}
♀ *Bx;; sqh-GFP* > ♂ *Ras*^{V12}

Fig. 5

A-E:
♀*Bx* > ♂*w*^{1118}
♀*Bx* > ♂ *w*^{1118}*; Idgf3*^{KD}
♀*Bx* > ♂ *Ras*^{V12}
♀*Bx* > ♂ *w*^{1118}*; Idgf3*^{KD}*; Ras*^{V12}

F-J:
♀ *Bx* > ♂ *w*^{1118}*;; Idgf3::GFP*
♀ *Bx;; αSpec*^{KD} > ♂ *w*^{1118}*; Idgf3::GFP*
♀ *Bx* > ♂ *w*^{1118}*; Idgf3::GFP; Ras*^{V12}
♀ *Bx;; αSpec*^{KD} > ♂ *w*^{1118}*; Idgf3::GFP; Ras*^{V12}

K-T:
♀ *Bx* > ♂ *w*^{1118}
♀ *Bx* > ♂ *w*^{1118}*, JNK*^{DN}
♀ *Bx;; Idgf3-OE* > ♂ *w*^{1118}
♀ *Bx;; Idgf3-OE* > ♂ *w*^{1118}*, JNK*^{DN}
♀ *Bx* > ♂ *Ras*^{V12}
♀ *Bx* > ♂ *w*^{1118}*, JNK*^{DN}*;; Ras*^{V12}
♀ *Bx;; Idgf3-OE* > ♂ *Ras*^{V12}
♀ *Bx;; Idgf3-OE* > ♂ *w*^{1118}*, JNK*^{DN}*;; Ras*^{V12}
♀ *Bx, mCD8::RFP-OE* > ♂ *w*^{1118}*, JNK*^{DN}*;; Ras*^{V12}

U-V:
♀ *Bx* > ♂ *w*^{1118}*; TRE.GFP/CyO.GFP*
♀ *Bx;; αSpec*^{KD} > ♂ *w*^{1118}*; TRE.GFP/CyO.GFP*
♀ *Bx* > ♂ *w*^{1118}*; TRE.GFP/CyO.GFP; Ras*^{V12}
♀ *Bx;; αSpec*^{KD} > ♂ *w*^{1118}*; TRE.GFP/CyO.GFP; Ras*^{V12}

Fig. S5

A-B''':
♀ *Bx* > ♂ *w*^{1118}
♀ *Bx* > ♂ *Ras*^{V12}

C-G:
♀ *Bx* > ♂ *w*^{1118}*;; Idgf3::GFP*
♀ *Bx;; αSpec*^{KD} > ♂ *w*^{1118}*; Idgf3::GFP*
♀ *Bx* > ♂ *w*^{1118}*; Idgf3::GFP; Ras*^{V12}
♀ *Bx;; αSpec*^{KD} > ♂ *w*^{1118}*; Idgf3::GFP; Ras*^{V12}

H:
♀ *Bx; Idgf3::GFP* > ♂ *w*^{1118}
♀ *Bx; Idgf3::GFP* > ♂ *w*^{1118}*; Pak*^{KD}
♀ *Bx; Idgf3::GFP* > ♂ *Ras*^{V12}
♀ *Bx; Idgf3::GFP* > ♂ *w*^{1118}*; Pak*^{KD}*; Ras*^{V12}

I-Q:
♀Bx > ♂w^{1118}
♀*Bx;; Idgf3-OE* > ♂ *w*^{1118}
♀*Bx* > ♂ *Ras*^{V12}
♀*Bx;; Idgf3-OE* > ♂ *Ras*^{V12}

R-AD:
♀ *Bx* > ♂ *w*^{1118}
♀ *Bx* > ♂ *w*^{1118}*; Idgf3*^{KD}
♀ *Bx* > ♂ *w*^{1118}*, JNK*^{DN}
♀ *Bx;; αSpec-OE* > ♂ *w*^{1118}
♀ *Bx;; αSpec-OE* > ♂ *w*^{1118}*; Idgf3*^{KD}
♀ *Bx;; αSpec-OE* > ♂ *w*^{1118}*, JNK*^{DN}
♀ *Bx* > ♂ *Ras*^{V12}
♀ *Bx* > ♂ *w*^{1118}*; Idgf3*^{KD}*; Ras*^{V12}
♀ *Bx* > ♂ *w*^{1118}*, JNK*^{DN}*;; Ras*^{V12}
♀ *Bx;; αSpec-OE* > ♂ *Ras*^{V12}
♀ *Bx;; αSpec-OE* > ♂ *w*^{1118}*; Idgf3*^{KD}*; Ras*^{V12}
♀ *Bx;; αSpec-OE* > ♂ *w*^{1118}*, JNK*^{DN}*;; Ras*^{V12}
♀ *Bx, mCD8::RFP-OE* > ♂ *w*^{1118}*, JNK*^{DN}*;; Ras*^{V12}

Fig. 6
B:
♀ *Bx* > ♂ *w*^{1118}
♀Bx > ♂ w^{1118}; CH3L1-OE
♀*Bx* > ♂ *w*^{1118}*; CH3L2-OE*

C-P:
♀ *Bx* > ♂ *w*^{1118}
♀Bx > ♂ w^{1118}; CH3L1-OE
♀*Bx* > ♂ *w*^{1118}*; CH3L2-OE*
♀*Bx* > ♂ *Ras*^{V12}
♀Bx > ♂ w^{1118}; CH3L1-OE; *Ras*^{V12}
♀*Bx* > ♂ *w*^{1118}*; CH3L2-OE; Ras*^{V12}
